# Intelligent Sensing and Adaptive Control for Low-Loss Soybean Harvesting: A Review of Multi-Scenario Technologies

**DOI:** 10.3390/s26206575

**Published:** 2026-10-16

**Authors:** Yapeng Wu, Rui Zhang, Xingjian Yang, Ruifan Tang, Jiewen Yang, Shiguo Wang, Zhong Tang

**Affiliations:** 1 School of Agricultural Engineering, Jiangsu University, Zhenjiang 212013, China; 3240306037@stmail.ujs.edu.cn (Y.W.);; 2 Xinjiang Academy of Agricultural and Reclamation Science, Shihezi 832000, China

**Keywords:** soybean harvesting, intelligent sensing, multi-source sensing, online quality monitoring, machine vision, light detection and ranging, adaptive control

## Abstract

Soybean harvesting is strongly affected by low pod position, lodging, pod shattering, moisture variation, and changing material flow, making stable low-loss operation difficult across diverse field conditions. This review synthesizes 179 core publications identified through structured literature searches and citation tracking, including 69 direct soybean studies, six adjacent-legume studies, and 104 cross-crop technology-transfer studies. The literature is organized around a sensing–decision–actuation–feedback chain covering harvest-loss monitoring, grain-quality assessment, crop and terrain perception, navigation, and adaptive regulation. Low-loss header, threshing, separation, and cleaning technologies are examined as the physical processes through which sensing and control affect harvest-quality outcomes. The synthesis shows that future progress requires robust multi-source sensing under dust, vibration, illumination variation, and crop occlusion; field-calibrated state estimation; coordinated multi-actuator closed-loop control; standardized operating and quality databases; and validation across cultivars, terrains, and cropping systems. Representative soybean studies reviewed here reported reductions of approximately 30.4% in grain breakage, 62.6% in harvest loss, and 50.0% in cleaning loss under their respective experimental conditions.

## 1. Introduction

### 1.1. Demand for Soybean Production and Technical Barriers in Harvesting

Soybean is an important oilseed and protein crop widely used for food, edible oil, and animal feed [1]. The global soybean-harvested area increased from approximately 23.2 million ha in 1961 to 143.2 million ha in 2024, when production reached about 397.7 million tons [2]. Improving mechanized-harvesting efficiency, reducing harvest loss, and preserving grain quality are therefore essential for increasing the effective soybean supply. However, soybean plants are characterized by low pod position, lodging and pod-shattering susceptibility, and impact-sensitive seeds, while crop conditions at maturity vary with environment and management. These features create losses and mechanical damage during cutting, feeding, conveying, threshing, separation, and cleaning. In particular, an excessively high cutter height can leave low-position pods unharvested, whereas an excessively low cutter height increases the risk of header–ground contact and soil intake [3]. The header must therefore balance close-to-ground cutting, stable feeding, and low-impact operation. Vibration-control studies in rapeseed further show that reducing cutterbar and header-frame vibration can decrease shattering-related loss, providing a transferable design reference for soybean headers [4]. Harvest loss is also affected by crop condition, field environment, machine condition, operating settings, and operational management [5]. Equipment requirements consequently differ between large level fields, hilly or fragmented plots, and intercropping systems, and the narrow optimum harvest window further complicates parameter matching. Harvesting too early increases threshing and drying loads, whereas delayed harvest increases pod shattering, disease risk, and quality deterioration [6].

**Figure 1:**
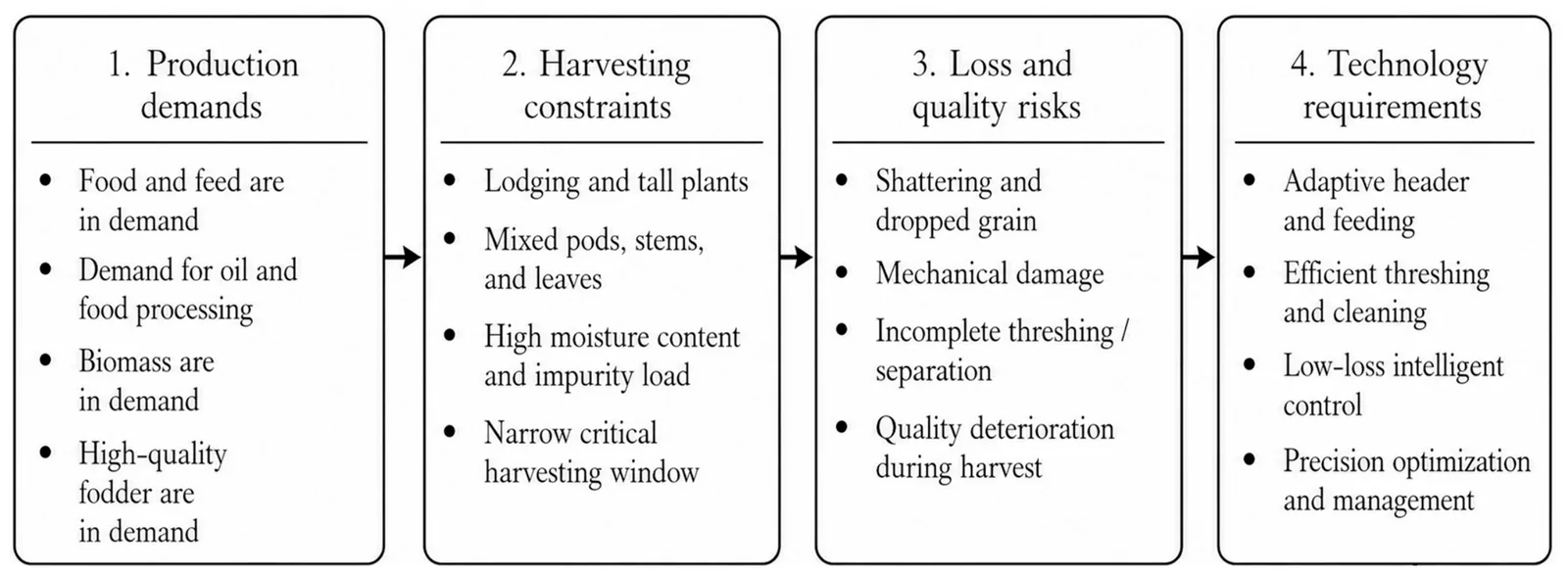
Relationships among production demands, harvesting constraints, loss and quality risks, and technological requirements for mechanized soybean harvesting. The arrows indicate the sequential relationship from production demands to harvesting constraints, resulting loss and quality risks, and corresponding technological requirements. Crop, machine, operating-parameter, and management conditions jointly influence these stages.

Current soybean-harvesting equipment remains constrained by insufficient crop-specific adaptation, experience-based parameter adjustment, and weak quality-monitoring and feedback mechanisms. Fixed settings cannot maintain stable performance when lodging severity, lowest pod height, moisture content, feed rate, and material composition change. Crop-row detection provides a sensing basis for row-following operation and header control [7]; online cleaning-quality monitoring can guide coordinated fan- and sieve-related adjustment [8]; moisture-dependent seed mechanics require threshing and conveying parameters to be matched to material state [9]; and adaptive, neural-network, and model-predictive control methods provide a basis for dynamic multi-parameter regulation [10]. Machine maintenance and operator proficiency remain important supporting factors [5]. Low-loss soybean harvesting should therefore be treated as an end-to-end quality-control problem that links crop and machine sensing, parameter decision-making, actuation, harvest-quality feedback, maintenance, and operation. Figure 1 summarizes the systemic challenges and corresponding technical requirements for low-loss, efficient, and adaptable mechanized soybean harvesting.

### 1.2. Related Works and Research Motivations

Research on mechanized soybean harvesting has progressed from traditional harvesting methods and isolated component improvements toward integrated systems encompassing low-loss headers, threshing and cleaning, grain handling, quality monitoring, navigation, and intelligent control [11]. Compatibility between cultivar traits and mechanical harvesting requirements is also important. Evidence from rapeseed shows that greater shattering resistance, compact plant architecture, and uniform maturity can reduce mechanical-harvesting loss, indicating that low-loss soybean harvesting also requires closer integration between machinery and agronomy [12]. Machine vision has enabled real-time classification of intact soybean grains, broken grains, and impurities, providing measurable feedback for harvest-quality assessment and operating-parameter adjustment [13]. Adaptive regulation has likewise been demonstrated in soybean cleaning and threshing: real-time cleaning-loss and impurity measurements can coordinate fan- and sieve-related settings [8], while feed-rate estimation from header height, moisture, and feeder-house images can drive dynamic adjustment of threshing and separation clearances [14]. Low-impact biomimetic mechanisms developed for other crops provide complementary principles for compliant feeding and threshing [15].

Together, these studies show a transition from fixed-parameter operation toward sensing-guided, coordinated regulation. Nevertheless, most evidence remains limited to particular cultivars, machine models, and operating environments, and cross-regional adaptability, interference resistance, and long-duration reliability require further validation. Coupled discrete-element and multibody-dynamics simulation is increasingly used to analyze crop–machine interactions and optimize structures and operating parameters [16], but methodological heterogeneity in crop-condition reporting, machine settings, and loss-measurement procedures still limits direct cross-study comparison [5]. Existing reviews cover soybean harvest-loss factors, equipment development, and broader legume mechanization [1,5,11], yet three gaps remain: direct soybean evidence and transferable cross-crop evidence are not always clearly distinguished; crop morphology, planting pattern, equipment structure, operating parameters, sensing, control, and harvest-quality outcomes are rarely integrated within one analytical framework; and most intelligent systems are validated only for individual components or operating conditions. These gaps motivate a review that explicitly classifies evidence levels and examines low-loss equipment, intelligent sensing and control, and multi-scenario adaptation as a connected system.

### 1.3. Overview Framework and Literature Scope of This Article

This review focuses on four linked domains: soybean crop and scenario constraints, low-loss harvesting equipment, intelligent sensing and adaptive control, and multi-scenario adaptation. Crop morphology, lowest pod position, moisture content, and material properties are included because they define feasible conditions for cutting, feeding, threshing, and cleaning [17]. Mechanical modeling and contact-damage analysis from related biological materials provide additional methodological support for low-loss component design [18]. Harvesting routes and low-loss components are then evaluated as the physical processes through which sensing and control affect harvest quality; transferable cleaning and intelligent-equipment studies are used only where the technical pathway can be mapped clearly to soybean harvesting [19,20].

This review also considers large-scale plain farming, strip intercropping, hilly and mountainous areas, and fragmented plots because these scenarios alter machine width, travel path, header configuration, feed load, and parameter requirements [21]. Soybean-specific studies on low-loss components are used to analyze losses caused by low pods, lodging, pod shattering, and mechanical damage [22], while systematic reviews are used to interpret interactions among crop condition, machine performance, parameter settings, and management [5]. Direct soybean evidence is distinguished from adjacent-legume and cross-crop technology-transfer evidence throughout the discussion.

**Figure 2:**
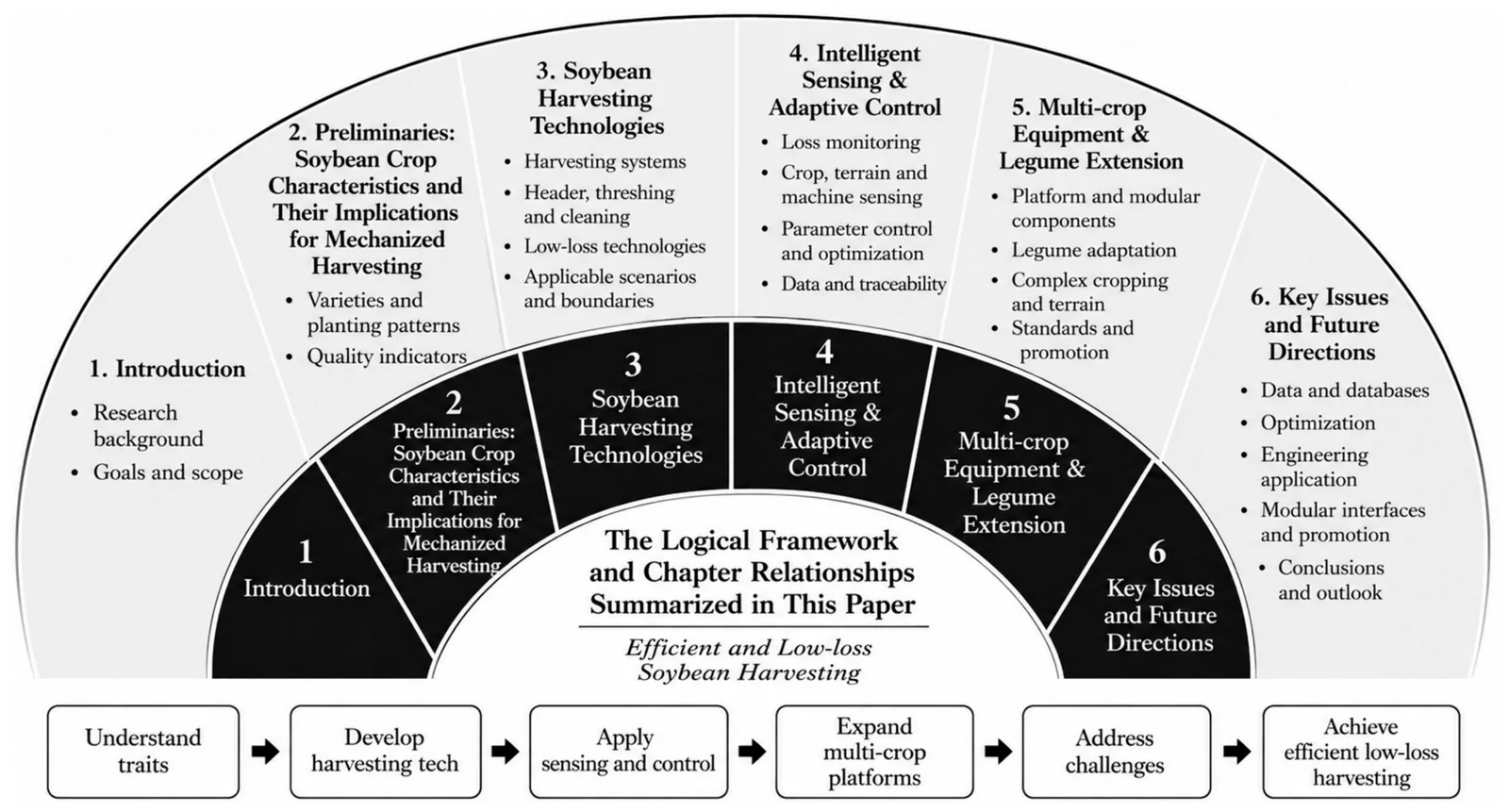
The logical framework and chapter relationships summarized in this paper. The black inner sectors represent the six main sections of the review, whereas the grey outer sectors summarize the principal contents of each section. The arrows at the bottom indicate the logical progression from understanding crop traits, through harvesting-technology development, sensing and control, and multi-crop platform expansion, to addressing technical challenges and achieving efficient low-loss soybean harvesting.

Figure 2 summarizes the logical framework of this review, linking crop and scenario constraints, loss-formation mechanisms, equipment structures and operating parameters, sensing and control, and harvest-quality outcomes. Within this framework, the present review addresses three research questions. RQ1: Which biological, agronomic, and machine-level factors limit stable low-loss soybean harvesting? RQ2: How do existing harvesting equipment, intelligent sensing methods, and adaptive-control strategies address these limitations, and what evidence supports their reported performance? RQ3: Which technical gaps and validation requirements constrain the reliable deployment of intelligent soybean-harvesting systems across different cultivars, terrains, and cropping systems?

Accordingly, this review makes three contributions relevant to intelligent sensing in precision agriculture. First, it distinguishes direct soybean evidence from adjacent-legume and cross-crop technology-transfer evidence so that sensor and control performance is interpreted within explicit applicability boundaries. Second, it organizes the literature as a scenario-constraints–sensing/state-estimation–equipment-parameter–control-action–harvest-quality-feedback chain, thereby linking physical harvesting processes with information acquisition and adaptive regulation. Third, it compares the technical boundaries of major production scenarios and identifies research priorities for multi-source sensing, AI-enabled state estimation, coordinated control, and cross-scenario validation.

### 1.4. Literature Search and Critical-Synthesis Approach

This study adopted a structured literature review and critical-synthesis approach to examine the principal biological, mechanical, sensing, and control processes involved in low-loss soybean harvesting. To improve the transparency of literature selection and reduce arbitrary evidence inclusion, predefined databases, search pathways, literature-selection criteria, evidence-classification rules, and thematic-synthesis procedures were applied. Because specialized soybean-harvesting technologies are closely related to general agricultural-equipment technologies, the literature was classified into direct soybean evidence, adjacent evidence from related legume species, and cross-crop technology-transfer evidence. Evidence categories were distinguished from validation levels. Soybean-specific supporting studies and transferable evidence from related legumes or other agricultural systems were used to establish engineering constraints and potential technical pathways, but they were not regarded as direct validation of improved soybean-harvesting performance.

(1) Data Sources and Search Strategy

Literature searches were conducted in the China National Knowledge Infrastructure (CNKI), Web of Science Core Collection (WoS Core Collection), and ScienceDirect databases. The search period extended from January 2000 to July 2026, with the final search performed on 27 July 2026; backward citation searching was used to identify seminal studies and representative engineering applications. Two complementary search pathways were adopted. The first focused on soybean-harvesting equipment, harvest loss, and material properties. In WoS, the query was as follows: TS = (“soybean” OR “Glycine max”) AND TS = (harvest* OR “combine harvest*” OR “combine harvester*” OR header* OR cutterbar* OR reel* OR thresh* OR separat* OR clean* OR “harvest loss*” OR “header loss*” OR “grain damage” OR “seed damage” OR shatter* OR lodging OR “cutting height”).

The 2000–2026 database window was applied to the electronic database searches. Earlier seminal studies were eligible when identified through backward citation tracking and when they provided foundational evidence directly relevant to soybean harvesting or material properties. Intelligence-related terms were not imposed as mandatory conditions for all records, because doing so would have excluded conventional harvesting-equipment, crop-mechanics, and material-property studies that define the physical processes and state variables ultimately targeted by intelligent sensing and adaptive control. The two search pathways were therefore designed to be complementary rather than mutually restrictive.

The second pathway focused on intelligent sensing, online monitoring, navigation, and adaptive control. The corresponding WoS query was as follows: TS = (“soybean” OR “Glycine max”) AND TS = (harvest* OR “combine harvester*”) AND TS = (sensor* OR monitor* OR “loss monitoring” OR “moisture monitoring” OR “feed-rate monitoring” OR “machine vision” OR LiDAR OR GNSS OR navigation OR “header height control” OR “header flotation” OR “terrain following” OR “adaptive control” OR “automatic control” OR “intelligent control” OR “model predictive control” OR “machine learning” OR “deep learning”).

Because the available search fields, indexing structures, and query syntax differed among the three databases, the search expressions were adapted accordingly, while maintaining the same thematic search logic.

Records retrieved through the two principal search pathways were manually exported and consolidated by two authors (Yapeng Wu and Rui Zhang). Duplicate records were identified and removed through manual examination of bibliographic information. The two authors jointly conducted title and abstract screening, followed by full-text assessment according to the predefined inclusion and exclusion criteria. Study inclusion and evidence classification were also performed manually by the two authors. No automated deduplication or literature-screening software was used. Because direct soybean evidence did not adequately cover all relevant technologies, targeted supplementary searches were conducted for clearly identifiable processes, including terrain-following headers, compliant contact, threshing and separation, cleaning control, navigation, communication interfaces, and modular equipment. These supplementary searches used combinations of “technical keyword + target crop or agricultural-equipment keyword”. For each included record, the source, rationale for inclusion, and correspondence to a specific soybean-harvesting stage were documented. Supplementary publications were classified according to their research objects and evidence roles. Soybean-specific studies addressing identifiable harvesting constraints were considered for the D2 supporting-evidence sublevel, whereas studies involving related legumes, other crops, or general agricultural machinery were classified as adjacent-legume or cross-crop technology-transfer evidence according to their technical applicability.

The search strategy initially retrieved 8669 records. After duplicate removal, 4413 remained; title and abstract screening excluded 3954 records, leaving 459 records for full-text assessment. Following the application of the eligibility and evidence-classification criteria, 179 peer-reviewed publications were retained for core thematic synthesis. These included 69 direct soybean publications (38.55%), six adjacent-legume publications (3.35%), and 104 cross-crop technology-transfer publications (58.10%).

**Figure 3:**
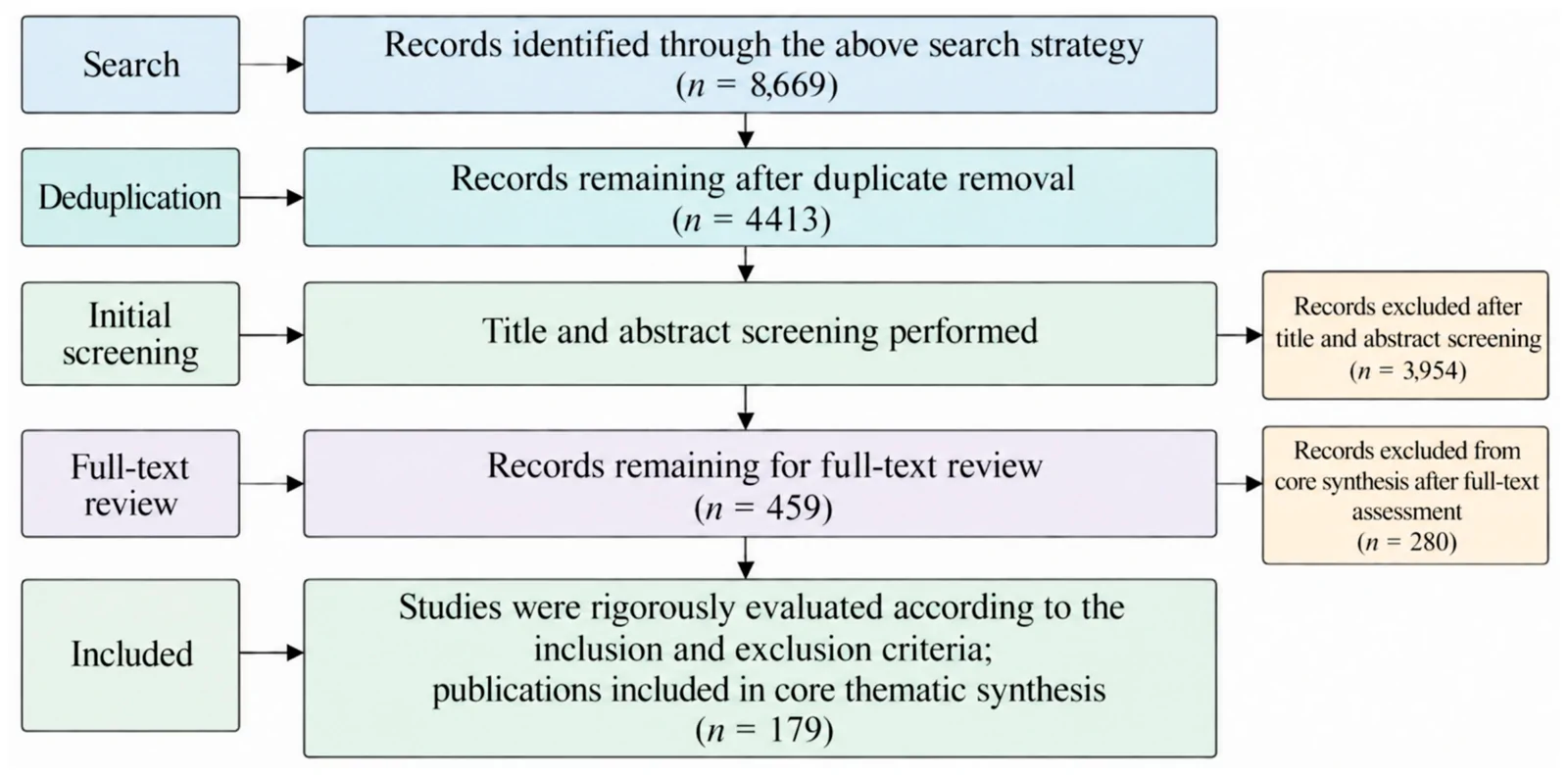
Literature identification and selection process. The arrows indicate the sequential literature-selection process, and the different fill colors distinguish the main screening stages, while the pale-yellow boxes indicate excluded records. Note: The flowchart presents the selection of publications for core thematic synthesis. Twenty additional peer-reviewed publications were cited for contextual or methodological purposes, and one FAOSTAT statistical database source was used for background production statistics. These sources were not included in the core evidence count.

The reference list contains 200 sources in total, comprising the 179 core publications, 20 additional peer-reviewed publications cited for contextual or methodological support, and one FAOSTAT statistical database source. The latter 21 sources were retained as supporting references but were not counted in the core thematic evidence synthesis. This distinction was maintained when interpreting evidence strength and drawing conclusions regarding soybean-harvesting performance. The literature identification and selection process is summarized in Figure 3.

(2) Literature Selection Criteria

The retrieved records were evaluated sequentially according to predefined inclusion and exclusion criteria and classified into three mutually exclusive evidence categories based on their research objects and relevance to soybean harvesting. Direct soybean evidence was further divided into two sublevels. The first (D1) comprised soybean-specific harvesting studies investigating harvest-loss mechanisms, machine components, sensing and control methods, or harvest-quality outcomes through field experiments, laboratory tests, simulations, or other technical evaluations. The second (D2) comprised soybean-specific supporting studies addressing crop morphology, material properties, moisture conditions, planting configurations, end-use quality requirements, and crop-zone equipment arrangements that could be explicitly linked to harvesting-machine design, parameter selection, or operational constraints. D2 studies were used to establish crop- and scenario-specific engineering requirements rather than to demonstrate harvesting-system performance. Adjacent-legume evidence included studies on related legume crops, such as peanut, chickpea, pea, and lentil, with comparable plant characteristics, seed-damage mechanisms, or harvesting processes. Cross-crop technology-transfer evidence included studies on other crops or general agricultural machinery that provided potentially transferable principles related to sensing, monitoring, terrain following, compliant contact, navigation, communication interfaces, or adaptive control. Such evidence was included only when a specific technical relationship to soybean harvesting could be identified, and validation in the source application was distinguished from validation in soybean harvesting.

In terms of research scope, eligible studies were required to contribute to at least one component of the scenario-constraints–sensing–decision–actuation–harvest-quality-feedback framework. Relevant topics included soybean harvest-loss mechanisms, low-loss harvesting equipment, crop and material characteristics affecting harvesting operations, optimization of threshing, separation, and cleaning processes, intelligent sensing and adaptive control, and equipment adaptation to different production scenarios. Soybean-specific studies of planting geometry, crop-zone protection, and machinery configuration were considered as supporting evidence when their findings could inform identifiable constraints on harvesting-machine layout or operation. For studies conducted outside harvesting operations, the applicability of the reported methods or engineering principles was explicitly qualified. Only peer-reviewed journal articles and other peer-reviewed academic publications were eligible for the core thematic synthesis.

Non-academic publications, including patent specifications, news reports, product manuals, technical white papers, and non-peer-reviewed online resources, were excluded from the core evidence synthesis. Peer-reviewed publications focusing exclusively on general crop cultivation, irrigation, fermentation, food processing, regional branding, or agricultural management were treated as contextual references when no explicit relationship to soybean-harvesting constraints or engineering functions could be established. Studies addressing soybean end-use quality requirements were eligible as D2 supporting evidence only when their findings could inform measurable harvest-quality objectives or equipment-related damage mechanisms. Similarly, soybean-specific studies involving non-harvesting field operations were considered for D2 classification only when they provided identifiable crop-zone, spatial-configuration, or equipment-layout constraints relevant to potential harvesting-machine adaptation. Such studies were not interpreted as evidence of improvements in soybean harvest loss, grain breakage, or harvesting-control performance. When substantially the same research was reported in multiple publications, the most complete version was prioritized. Studies were excluded from core synthesis when the harvesting-related technical relevance was insufficiently established or when the available evidence could not support meaningful thematic analysis.

(3) Literature Analysis and Thematic Synthesis

For each included study, core information—including the research question, principal findings, experimental conditions, and technical parameters—was extracted and then classified, synthesized, and compared according to the logical structure of this review. On this basis, the technological development trajectory, areas of academic consensus, points of debate, current knowledge gaps, and future research challenges were identified to support the subsequent discussion.

To distinguish evidence relevance from validation strength, the included publications were evaluated according to their research objects, experimental conditions, completeness of crop- and machine-condition reporting, replication and statistical analysis, field validation, and applicability to soybean harvesting. Within the direct soybean category, D1 studies were treated as harvesting-specific evidence, whereas D2 studies were treated as crop-, material-, quality-, or scenario-related supporting evidence. Adjacent-legume and cross-crop studies were assessed according to the comparability of their investigated mechanisms, equipment functions, and operating conditions with soybean harvesting. Primary experimental studies and secondary reviews were distinguished to avoid interpreting repeated reporting of the same findings as independent experimental validation. Quantitative conclusions concerning soybean-harvesting performance were based on soybean-specific harvesting studies, while D2, adjacent-legume, and cross-crop evidence was used primarily to explain engineering constraints, transferable principles, and research gaps.

The remainder of this review is organized as follows. Section 2 introduces soybean crop characteristics, planting patterns, and harvest-quality requirements. Section 3 reviews soybean-harvesting methods and low-loss harvesting equipment. Section 4 synthesizes intelligent sensing, navigation, and adaptive-control technologies. Section 5 examines multi-crop equipment platforms, scenario-specific adaptation, and deployment constraints. Finally, Section 6 discusses unresolved technical challenges and future research directions for intelligent low-loss soybean harvesting.

## 2. Preliminaries: Soybean-Crop Characteristics and Their Implications for Mechanized Harvesting

### 2.1. Soybean Types and Material Properties During Different Maturity Stages

Soybeans can be broadly classified into grain-type and food-type categories according to end use, seed characteristics, and processing requirements. These types differ in nutritional composition, flavor, seed size, and processing performance, leading to different requirements for quality preservation during harvesting, handling, and storage [23]. Seed composition is also associated with rupture force, elastic modulus, and deformation behavior, while moisture content further alters mechanical response and susceptibility to damage [24]. At maturity, soybean commonly exhibits low pod position, heterogeneous stem conditions, lodging, pod-shattering susceptibility, and variable seed moisture. First-pod height directly determines whether low-position pods can be recovered effectively and is governed by main-stem node number, internode length, and the position of the first pod-bearing node [25], while management and environment also influence lowest-pod height [26]. These traits define the feasible range of cutterbar height and the risk of missed harvesting, ground contact, and soil intake.

**Figure 4:**
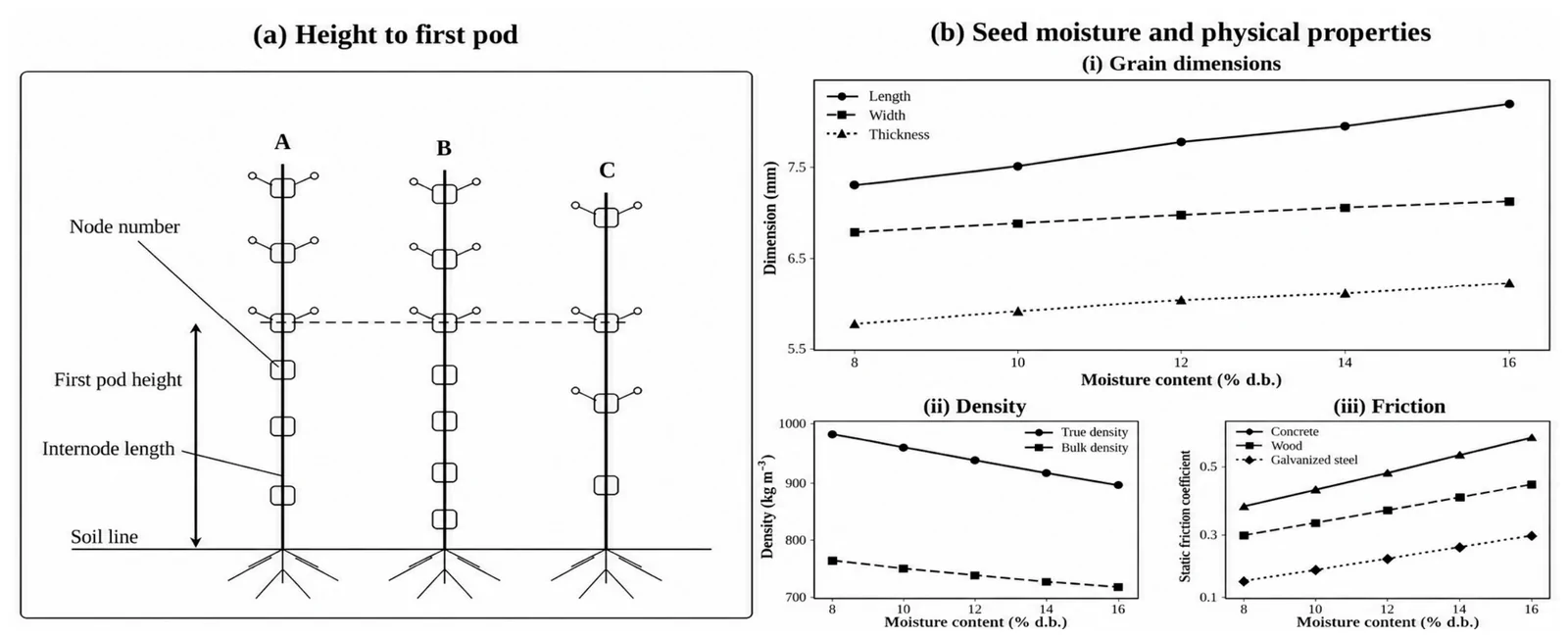
Plant architectural determinants of first pod height and moisture-dependent physical properties of soybean seeds. (**a**) Effects of node number and internode length on first pod height [25]: A, regular nodes and internodes; B, a greater number of nodes with shorter internodes; C, fewer nodes with longer internodes. The dashed line indicates the same height reference for first pod position among the three schematic plants. (**b**) Moisture-dependent physical properties of soybean seeds [27]: (**i**) changes in grain dimensions (length, width, and thickness) with increasing seed moisture content; (**ii**) changes in true density and bulk density with increasing seed moisture content; and (**iii**) changes in the static friction coefficient on concrete, wood, and galvanized steel with increasing seed moisture content.

Seed moisture content is another major determinant of physical properties and mechanical damage. Over a moisture range of 8–16%, soybean seed dimensions increase, density generally decreases, and the static coefficient of friction rises [27], affecting motion, contact, and separation during threshing, conveying, and cleaning. Transferable DEM studies on geometrically complex crop materials show that realistic particle geometry and calibrated contact parameters are necessary for reliable simulation [28]. Figure 4 summarizes the two principal groups of constraints considered here: plant-architectural determinants of first-pod height and moisture-dependent soybean-seed properties. As shown in Figure 4, first-pod height defines the lower geometric boundary for header operation, whereas moisture-dependent changes in dimensions, density, friction, and mechanical response influence downstream threshing, conveying, and cleaning. These variables should therefore be treated as linked inputs to equipment design, numerical simulation, and operating-parameter adjustment.

Beyond low pod position, mature soybean may exhibit lodging, extensive branching, uneven maturity, and strong susceptibility to pod shattering. Stem strength, plant height, branching pattern, and overall architecture are therefore important indicators of mechanical-harvesting suitability [29]. Although not soybean-specific, biomimetic harvesting studies show that compliant contact and coordinated plant handling can balance harvesting efficiency with loss reduction [30], providing transferable design principles for crop-lifting and flexible-feeding components used with lodged soybean.

Harvesting behavior is ultimately governed by the combined effects of plant architecture, pod distribution, stem condition, maturity uniformity, and seed moisture. Low pod position and lodging increase missed cutting and feeding instability, whereas low moisture increases pod dehiscence, grain shedding, seed-coat cracking, and breakage [25,31]. High moisture makes stems and pods tougher and increases incomplete threshing, entanglement, and cleaning load. The header should therefore provide close-to-ground terrain following, lodged-crop lifting, and compliant feeding, while threshing, conveying, and cleaning parameters should respond to moisture, feed rate, and material composition. Multi-input–multi-output modeling has shown that intelligent algorithms can support coordinated adjustment of concave clearance, cylinder speed, fan speed, and sieve opening [32]. Thus, soybean end use and mature-crop material properties jointly define the biological and mechanical basis for the equipment-matching and adaptive-control requirements discussed below.

### 2.2. Requirements for Matching Planting Methods with Machinery

The applicability of soybean-harvesting equipment depends on planting system, row spacing, strip width, field size, terrain, and crop condition in addition to machine structure and operating parameters. Row spacing should be coordinated with machine track width, header width, and traffic lanes [33]. A multi-criteria selection model tested on ploughs and rotary cultivators in a wheat–maize system [34] offers a machinery-matching method, although its applicability to soybean harvesters remains untested. Soybean may be cultivated as a monocrop or within double-cropping, relay-cropping, and maize–soybean intercropping systems [35]. Under monocropping, equipment matching mainly concerns close-to-ground cutting, stable feeding, and suitable threshing and cleaning settings; even here, cleaning performance depends on coupled feed rate, material distribution, fan speed, and sieve opening [36]. Relay and strip intercropping alter crop spatial arrangement, row geometry, plant density, and harvesting paths and therefore impose additional requirements on machine configuration [37].

Intercropping equipment must support crop-row recognition, strip matching, selective harvesting, adjacent-crop protection, and stable feed loading. Crop-specific headers should match row spacing, strip width, and target-crop position [38], while intelligent intercropping frameworks based on row recognition, target identification, path decision-making, and execution control provide a basis for selective harvesting and travel-path regulation [39]. Variations in plant density and water availability further change crop growth and feed load [40]. These effects propagate beyond the header by altering straw-to-grain ratio, lateral material distribution, threshing load, sieve-layer thickness, and airflow demand. Reliable crop-row recognition is therefore important for row following and effective-width control [7]. In hilly terrain, trafficability and attitude stability become equally important; adjustable crawler-chassis systems provide an engineering reference for ground-clearance and attitude regulation [41]. Overall, monocropping emphasizes low-loss continuous harvesting, intercropping emphasizes strip recognition and selective operation, and hilly or fragmented fields require compact, maneuverable, terrain-adaptive machinery. Equipment matching should therefore be based on an integrated planting-system–crop-state–machine-structure–control framework.

### 2.3. End-Use Requirements and Harvest-Quality Control Indicators

Harvest-quality objectives should be differentiated by soybean end use because commercial grain, seed, food-processing, and fresh vegetable soybean are harvested for different downstream functions. The relative importance of harvest loss, grain breakage, impurity content, seed or pod integrity, germination, vigor, sanitary quality, and market appearance therefore differs among categories [11]. For commercial grain soybean, the principal objective is to maximize recoverable yield while maintaining market grade and storage safety. Total loss, header loss, threshing and cleaning loss, grain breakage, and impurity content are the main indicators, and soybean adaptive-cleaning studies show that real-time feedback can coordinate cleaning settings to reduce both cleaning loss and impurity [8]. Commercial-grain harvesting should therefore balance low loss, low breakage, and low impurity rather than optimize a single indicator.

Seed soybean requires a stricter definition of mechanical damage because visible breakage does not capture latent seed-coat, cotyledon, or embryonic injury. Mechanical impact can reduce viability and seedling performance [42], while visible-light and X-ray imaging provide additional means of assessing physical purity, viability, and vigor [43]. Harvest delay also reduces physiological and sanitary seed quality [6]. Seed–soybean control should therefore combine timely harvesting, gentle threshing, low-impact conveying, reduced drop height, and appropriate quality assessment. Electronic-nose studies in food systems illustrate transferable approaches for rapid sanitary screening [44,45], but these methods are treated here only as background methodology rather than direct harvester-control evidence.

Food-processing soybean places greater emphasis on grain integrity, size uniformity, seed-coat condition, cleanliness, and processing suitability [23]. Cracked soybean can deteriorate more rapidly during storage and reduce tofu yield [46], supporting the need for gentle threshing, low-impact conveying, fine cleaning, and, where necessary, grading and batch segregation. Fresh vegetable soybean differs because it is marketed primarily as intact pods; pod appearance, color, cracking, shattering, abrasion, and bruising become direct quality attributes, and the appropriate harvest window occurs before pronounced pod yellowing [47]. Closed-loop perception-and-execution methods developed for harvesting robots provide a transferable reference for pod recognition, path planning, and compliant low-damage handling [48].

These end-use differences should ultimately be translated into measurable quality indicators and adjustable machine parameters. Header height, forward speed, reel speed, cylinder or rotor speed, concave clearance, fan speed, and sieve opening dominate grain-oriented harvesting, whereas seed and fresh-market applications place additional emphasis on reduced impact, lower drop height, compliant contact, and gentle handling. Table 1 summarizes the relationship among end-use category, quality indicators, harvesting risks, control priorities, major equipment stages, and supporting evidence. The table is intended as a qualitative synthesis rather than a numerical ranking, because crop type, moisture content, machine configuration, and evaluation methods differ among studies.

As shown in Table 1, commercial grain soybean is controlled primarily through loss, breakage, and impurity indicators across the header, threshing, separation, and cleaning systems. Seed soybean adds germination, vigor, internal damage, and sanitary quality, shifting emphasis toward timely harvesting and low-impact handling; food-processing soybean requires high seed integrity, size uniformity, and cleanliness; and fresh vegetable soybean prioritizes intact pods and marketable appearance. Nutritional composition, flavor, and pathogen contamination are also influenced by cultivar, storage, and processing and are therefore treated as end-use attributes rather than direct harvester outputs. Future systems should establish an end-use-oriented chain linking use objective, measurable quality indicator, state sensing, operating parameter, and intelligent regulation so that machine settings can be adjusted according to both real-time material condition and product-quality requirements.

### 2.4. Effects of Low Pod Height, Lodging, Pod Shattering, and Moisture Content

Low pod position, lodging, pod shattering, and moisture variation are the principal crop-level determinants of soybean harvest loss and mechanical damage. Low pods increase missed harvesting as cutting height rises [17], while terrain-following headers can reduce low-pod loss under uneven-ground conditions [22]. Reel speed must also be matched to forward speed and crop condition because excessive contact promotes pod shattering whereas insufficient reel action causes missed or unstable feeding [50]. Lodging further increases crop pushing, entanglement, missed cutting, and non-uniform feeding; field studies show that lodging increases combine-harvesting loss and that loss rises further with cutting height [51]. Rainfall and harvest delay can simultaneously alter plant posture, seed moisture, disease incidence, and shattering risk [52], emphasizing the need to coordinate machine operation with timely harvest.

Pod shattering is controlled by cultivar genetics, pod structure, temperature, humidity, and repeated wetting–drying cycles [53], and multi-environment studies confirm that resistance involves multiple loci and genotype–environment interactions [54]. Transferable rapeseed evidence likewise indicates that shattering resistance, plant architecture, and maturity uniformity should be considered together when evaluating mechanical-harvesting suitability [12]. Moisture content has a dual effect: low moisture increases brittleness, shattering, seed-coat cracking, and breakage, whereas high moisture increases incomplete threshing, entanglement, and cleaning load. The unthreshed-grain rate rises with pod moisture even as seed damage may decrease [55], so cylinder speed must balance threshing completeness and impact damage. A moderate soybean-seed moisture range can reduce crushing risk; however, the optimum depends on cultivar, maturity, and machine configuration [56]. Low-impact biomimetic threshing in other shattering-prone crops provides a transferable design reference under dry conditions [15].

**Figure 5:**
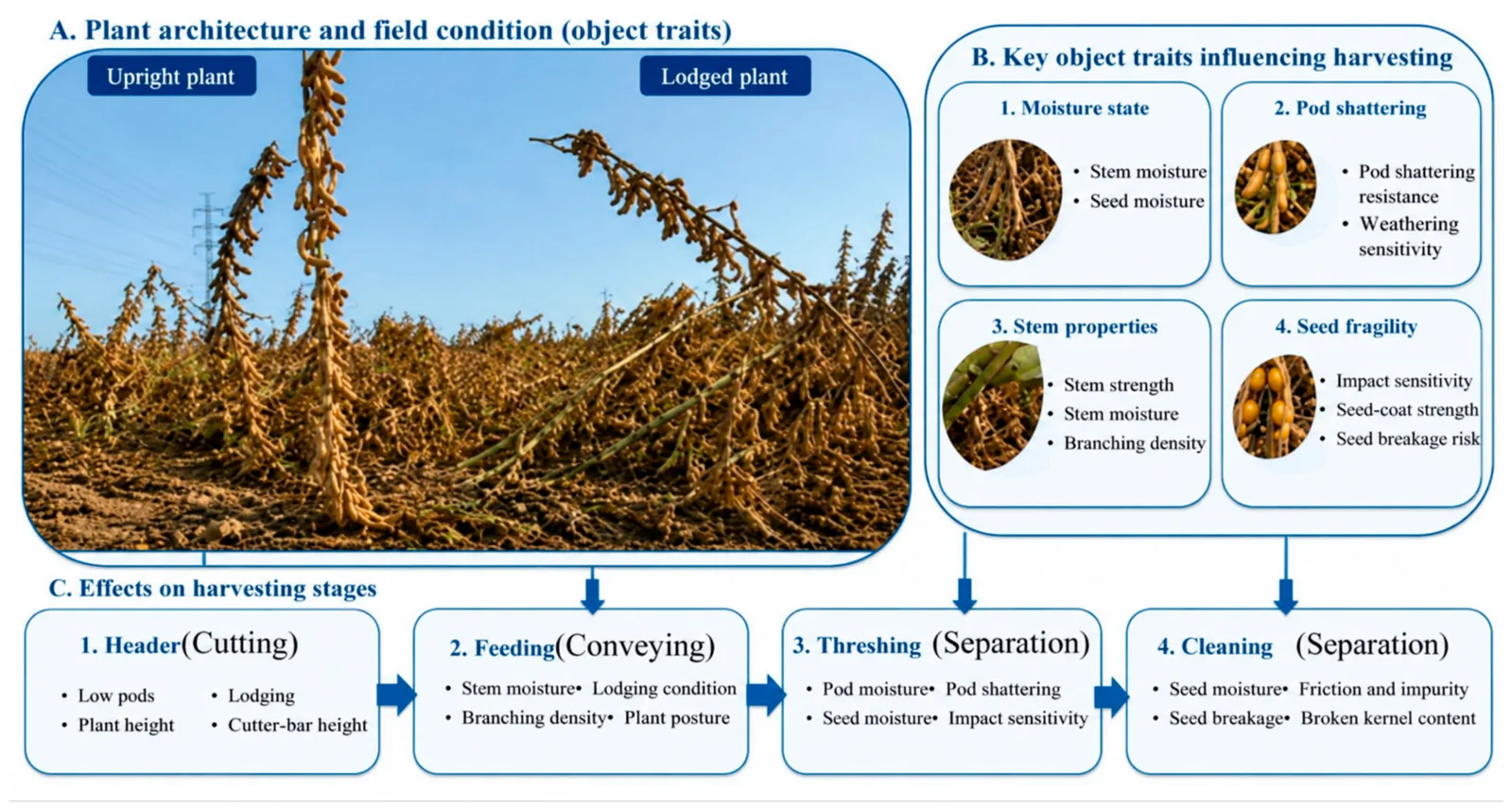
Conceptual relationships among mature-soybean plant architecture, material properties, and key combine-harvester stages. (**A**) Plant architecture and field conditions, illustrated by upright and lodged soybean plants. (**B**) Key object traits influencing harvesting, including moisture state, pod-shattering characteristics, stem properties, and seed fragility. (**C**) Effects of these crop and material traits on the main harvesting stages, including header cutting, feeding and conveying, threshing and separation, and cleaning and separation. The vertical arrows indicate the influence of plant architecture and object traits on the corresponding harvesting stages, whereas the horizontal arrows indicate the sequential material flow through the harvesting process from header cutting to cleaning. Blue text is used to highlight panel and harvesting-stage headings, while black text presents the corresponding descriptive information; the font colors are used only for visual distinction and do not represent quantitative categories.

These crop factors affect successive harvesting stages in different ways: low pod position and lodging primarily influence cutting and feeding, whereas pod shattering, seed fragility, stem condition, and moisture propagate into threshing, separation, and cleaning. The relationships are summarized in Figure 5.

As shown in Figure 5, the effects are coupled rather than independent. Lodging can increase both the amount and lateral non-uniformity of incoming material, while high stem moisture can further increase threshing and cleaning loads. Low-loss soybean harvesting consequently requires coordinated close-to-ground terrain-following cutting, lodged-crop lifting, appropriate reel-speed matching, compliant gathering and conveying, and moisture-adaptive threshing and cleaning. These engineering measures should be combined with timely harvesting and suitable cultivars. Future work should quantify the relationships among crop architecture, moisture state, feed load, machine settings, and harvest-quality indicators so that header height, reel speed, threshing intensity, and cleaning parameters can be regulated using real-time crop-condition and material-flow sensing.

### 2.5. Relationship Between Crop Characteristics, Cultivation Patterns, and Equipment Requirements

Equipment design and parameter selection should jointly consider planting geometry and crops’ material characteristics. Row spacing, strip width, plant density, and field layout determine header width, reel configuration, and travel path, whereas lowest pod height, stem condition, straw-to-grain ratio, and moisture content define suitable ranges for cutting, threshing, and cleaning. Studies of maize–soybean strip systems describe effects of planting configuration and interspecific resource competition on soybean growth and resource use [57,58]. However, their implications for canopy geometry and pod-height distribution during harvesting require direct field measurement; conventional monocrop equipment may therefore be unsuitable where crop geometry differs substantially [38]. Variable crop distribution also causes feed-rate fluctuations. Automatic reel-speed control can match reel action to forward speed and crop condition [59], while integrated crop-specific header modules on a common frame demonstrate one route toward coordinated harvesting in strip systems [60]. The 4GS-457 soybean header further shows that stable tine attitude and compliant reel action are valuable for reducing impact on low-position pods [61].

**Figure 6:**
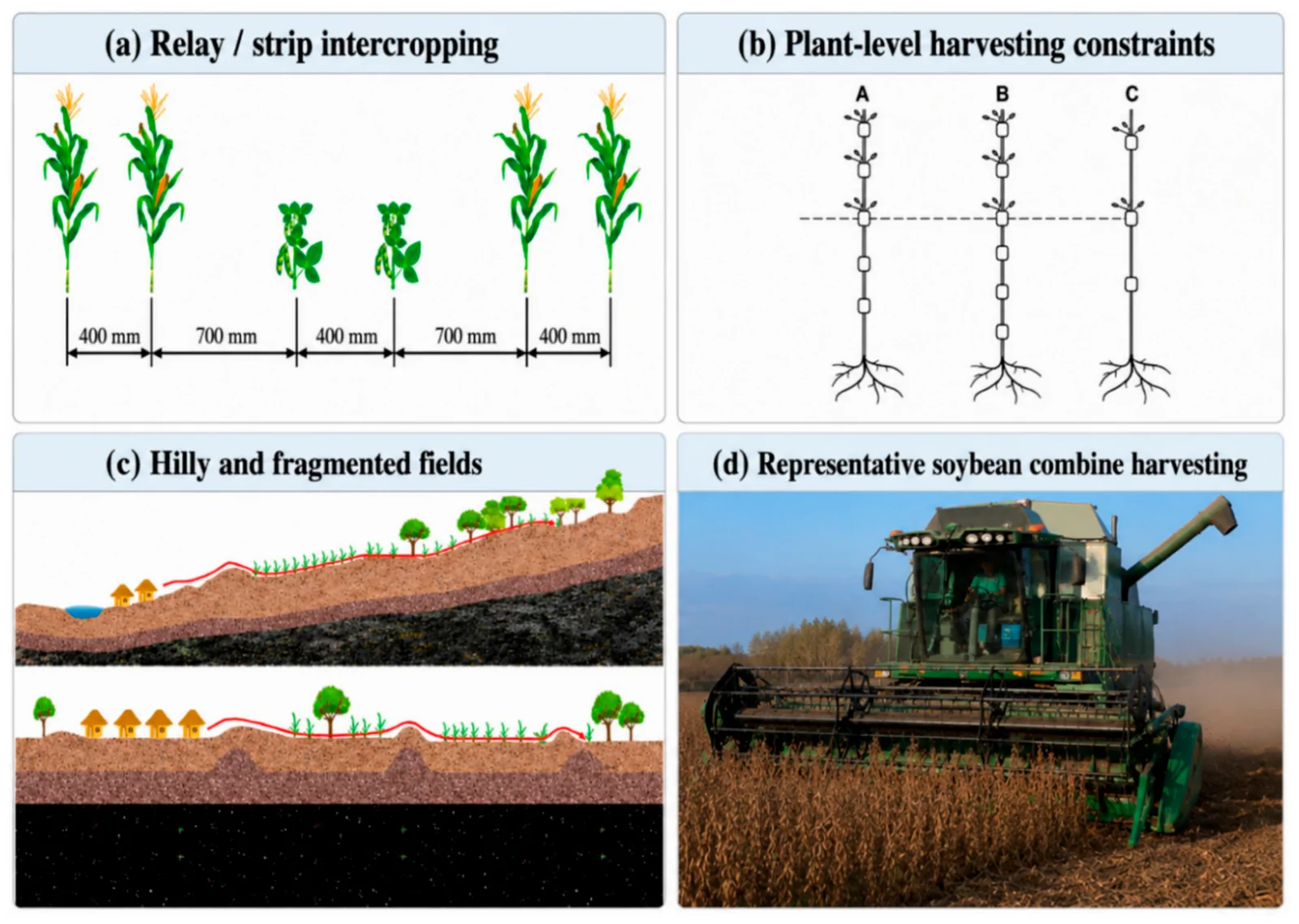
Representative soybean planting patterns, crop- and terrain-related harvesting constraints, and corresponding machinery-adaptation requirements. (**a**) Relay/strip intercropping configuration [22], with the arrows indicating the planting spacings; (**b**) plant-level harvesting constraints associated with first-pod height and plant architecture, where A represents regular nodes and internodes, B represents a greater number of nodes with shorter internodes, C represents fewer nodes with longer internodes, and the dashed line indicates the same reference height for first-pod position; (**c**) hilly and fragmented field conditions; and (**d**) representative soybean combine harvesting as an equipment-level example of machinery adaptation. Panels (**a**–**c**) were adapted from Nie et al. [22], Kuzbakova et al. [25], and Su et al. [65], respectively. Panel (**c**) illustrates terrain and parcel-access constraints rather than validation of harvester navigation.

Stem properties and straw-to-grain ratio further influence threshing and cleaning load. Transferable studies show that stem biomass and tissue condition vary with cultivar and growth stage [62], while soybean studies indicate that a high straw-to-grain ratio can impair threshing and increase sieve load [63]. Cylinder speed, concave clearance, airflow, and sieve opening should therefore respond to real-time material condition; adaptive soybean cleaning provides direct evidence for quality-feedback regulation [8]. Crop-state recognition can additionally support parameter presetting by linking cultivar, maturity, and moisture status to equipment databases; hyperspectral classification in other crops provides a transferable methodological reference [64]. Figure 6 summarizes how planting pattern, plant traits, terrain, and representative machinery jointly define adaptation requirements.

As shown in Figure 6, strip or relay planting requires crop-strip recognition and selective operation, while low pod position calls for close-to-ground cutting and header-height control. In fragmented or sloping landscapes, parcel geometry, road accessibility, and terrain slope are planning constraints [65]; their use for soybean-harvester routing and machine matching remains a proposed application rather than a result validated by Su et al. Whole-machine configuration must therefore account for crop and terrain constraints [22,25]. Planting systems and crop characteristics therefore define the design boundaries of soybean-harvesting equipment. Future harvesters should coordinate header, conveying, threshing, and cleaning systems within a unified matching framework that incorporates plant architecture, stem condition, moisture, feed load, terrain, and planting geometry and supports modular structures, crop-state recognition, and adaptive parameter regulation.

## 3. Soybean-Harvesting Methods and Low-Loss Equipment Technologies

**Figure 7:**
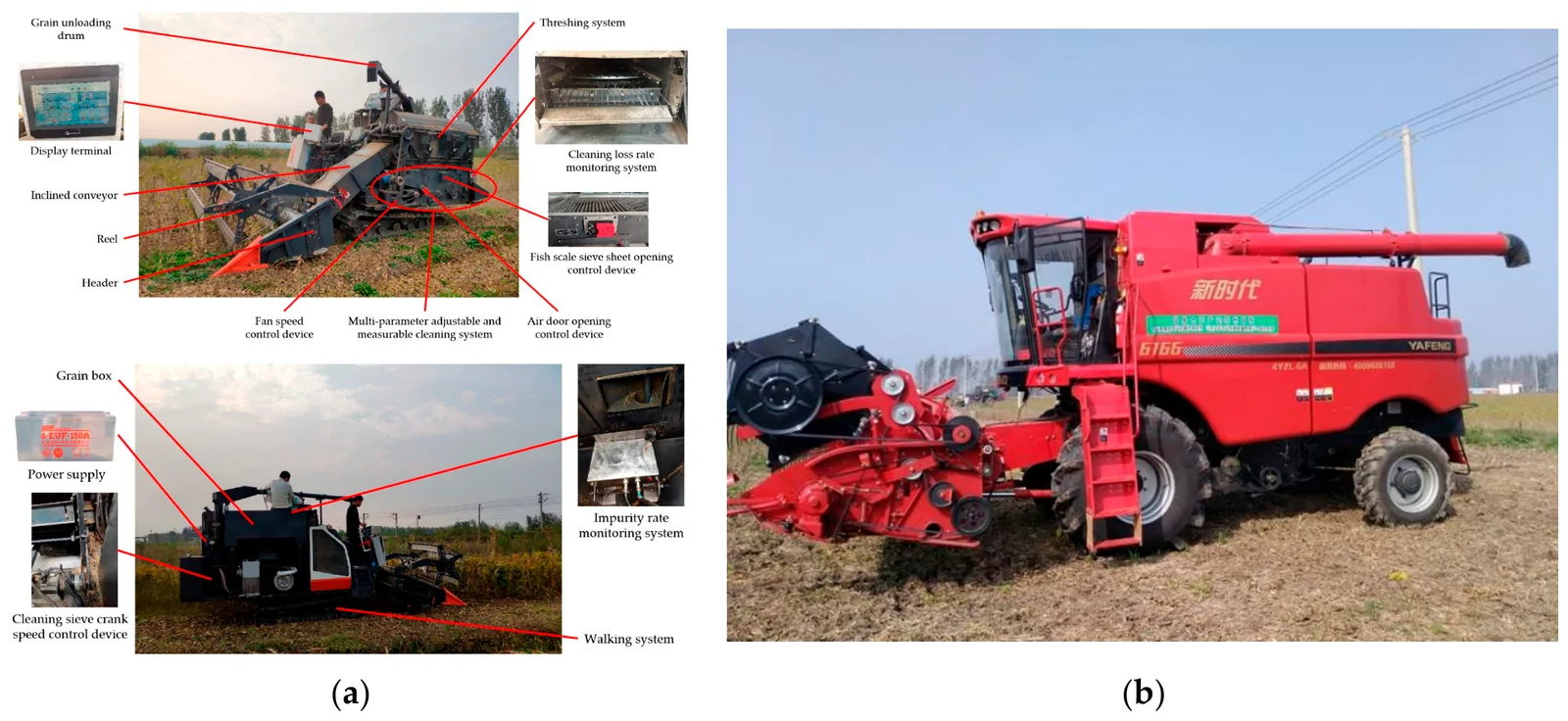
Representative combine-harvester platforms and intelligent systems relevant to soybean harvesting. (**a**) Soybean combine harvester equipped with an adaptive cleaning system and associated monitoring and control components, adapted from Liu et al. [8], Figure 4, under the Creative Commons Attribution (CC BY) license; (**b**) a conventional combine harvester shown as an example of a potentially adaptable multi-crop harvesting platform. The non-English text visible on the machine body in panel (**b**) consists of the original manufacturer branding and product/model markings and does not represent scientific annotation or convey additional scientific information in this figure.

Modern soybean harvesting increasingly integrates conventional combine-harvesting functions with sensing, navigation, and automated operation. A combine harvester coordinates cutting, feeding and conveying, threshing and separation, cleaning, and grain collection within a single harvesting platform, while GPS- and sensor-based autonomous operation can further improve field-operation precision and reduce labor requirements [66]. Representative combine-harvester platforms relevant to soybean harvesting are shown in Figure 7.

### 3.1. Roles and Applicability of Manual and Two-Stage Harvesting

Two-stage harvesting differs fundamentally from direct combine harvesting. Mature soybean plants are first cut and laid in windrows for field drying and are subsequently picked up, threshed, separated, and cleaned after stem and pod moisture contents have declined to an appropriate level. Although manual cutting and field drying require substantial labor and provide limited field capacity, they remain relevant for crops with very low pods, quality-sensitive production, and fields with restricted machine access, while also providing a useful benchmark for parameter selection and loss analysis in mechanized two-stage harvesting. The principal advantage of this method is its ability to regulate material condition before threshing. Direct combine harvesting requires the crop to satisfy the simultaneous requirements of cutting, feeding, conveying, threshing, separation, and cleaning within a narrow harvest window, whereas two-stage harvesting divides the process into cutting, field drying, and pickup threshing, allowing moisture and maturity conditions to be adjusted before the crop enters the threshing system. Studies of other shattering-prone crops have similarly identified trade-offs among field loss, operating cost, and weather exposure when comparing windrowing with direct combining, indicating that harvesting-method selection should account for the available weather window and the organization of field operations [67].

Regulation of crop moisture before threshing can improve both seed quality and storage suitability. Samarah et al. found that harvesting soybean at an earlier maturity stage and drying the seed within intact pods preserved germination and seed vigor more effectively than drying after pod removal [68]. Drying within the pod not only reduces moisture content but also moderates the rate of seed moisture loss, thereby supporting after-ripening and creating favorable conditions for low-damage threshing. Controlled drying can further supplement natural field drying after cutting. Using rice as the study material, Wang et al. combined pulsed-light treatment with thermal holding to achieve simultaneous drying and microbial inactivation [69]. Although this process was not developed specifically for soybean harvesting, it provides a transferable reference for low-temperature drying and sanitary-quality management of commercial and food-processing soybean. For soybean with low pods or prostrate plants, conventional combine headers may be unable to cut sufficiently close to the ground without increasing soil intake, whereas manual cutting or mechanized windrowing can generally achieve a lower cutting height. Changjai et al. reported that soybean seed obtained through manual cutting, field drying, and subsequent threshing had a lower moisture content than seed harvested directly by combine, suggesting that close-to-ground cutting and pre-threshing drying can reduce low-pod loss and improve storage suitability [70].

In seed production and other quality-sensitive applications, the major concern is mechanical damage during pickup, threshing, and conveying. Threshing impact can cause seed-coat cracking, cotyledon splitting, and latent internal damage. Arief et al. compared manual and mechanical threshing and found that mismatches among cylinder speed, seed size, and material condition intensified collision damage [71]. Threshing parameters following two-stage harvesting should therefore be matched to crop moisture state and mechanical properties rather than selected solely to maximize threshing completeness. Mechanical damage also depends on contact geometry and load distribution. Using apple gripping as the study process, Ji et al. showed that curved contact surfaces reduced local stress concentration and internal deformation [18], providing a methodological reference for compliant pickup, low-impact threshing, and buffered conveying components in two-stage soybean harvesting. Carvalho and Novembre further found that manual soybean harvesting preserved physiological seed quality over a wider moisture-content range than mechanical harvesting [72]. This finding indicates that two-stage harvesting can broaden the workable moisture window by regulating crop condition through field drying, thereby reducing both incomplete threshing at high moisture content and seed breakage at low moisture content.

Overall, two-stage and direct combine harvesting differ in harvest loss, seed quality, and adaptability to moisture variation. Changjai et al. reported lower total loss after manual cutting, drying, and threshing than after direct combine harvesting, primarily because of the lower cutting height and more uniform drying [70]. Arief et al. found that manual threshing produced a higher proportion of intact seed and less mechanical damage [71], while Carvalho and Novembre demonstrated the greater moisture-regulation capacity of non-direct harvesting [72]. Research on maize and soybean further indicates that harvest timing should balance latent dry-matter loss, seed moisture content, and physiological quality rather than rely solely on visible maturity [73]. These findings have three implications for mechanized two-stage harvesting. First, windrowers should provide a low and stable cutting height together with effective ground following to reduce losses from bottom pods [70]. Second, threshing quality depends on coordinated matching among cylinder speed, concave clearance, seed moisture content, and contact mode, and complete threshing should not be pursued merely by increasing cylinder speed. Pacheco et al. evaluated soybean seed quality under different threshing structures, cylinder speeds, concave clearances, and moisture conditions and found that higher cylinder speed aggravated mechanical damage, with injury severity varying according to seed moisture content [74]. These results confirm that post-windrow threshing settings should be adjusted jointly according to material moisture and device configuration. Third, two-stage harvesting remains technically relevant for seed soybean, high-quality food soybean, cultivars with very low pods, lodged crops, and hilly or fragmented fields. Its future development should progress from labor-intensive cutting and handling toward the mechanized integration of low cutting, uniform windrow formation, pickup feeding, compliant threshing, and controlled drying. Direct combine harvesting remains preferable for large, level fields, whereas two-stage harvesting can be selected in quality-sensitive or complex environments to balance loss reduction, field efficiency, and seed quality.

### 3.2. Current Status and Challenges of Direct Combine Harvesting

Direct combine harvesting is the dominant method in large-scale soybean production. A combine harvester completes cutting, feeding and conveying, threshing, separation, and cleaning in a single field pass and directly delivers cleaned grain. Compared with two-stage harvesting, it provides greater field capacity and requires less labor; however, crop conditions, field conditions, and all functional components must remain appropriately matched within the limited optimum harvest window. Insufficient adaptability at any stage can result in cumulative losses and quality deterioration. Soybean combine-harvesting loss consists primarily of header loss, threshing and separation loss, and cleaning loss, with header loss generally representing the largest component. Carreira et al. noted that crop-condition records, machine-parameter descriptions, and loss-measurement methods remain inconsistent among studies [5]. Older machines tend to produce greater losses, whereas forward speeds above 5 km/h do not necessarily increase loss, indicating that machine condition and parameter matching may be more influential than forward speed alone. Field measurements in Brazil showed that total combine-harvesting loss accounted for approximately 1.2–5.5% of soybean yield and that header loss was the dominant source, further supporting the prioritization of header-loss reduction rather than indiscriminate increases in threshing intensity [75].

The effect of forward speed is also conditioned by header type, feed rate, and material-flow continuity. Cortez et al. compared draper and auger headers and observed different loss responses across forward speeds, indicating that forward speed should be coordinated with header configuration and conveying stability [76]. Other studies have similarly shown that header type and forward speed interact to determine the stability of soybean harvest loss and should therefore be evaluated as coupled parameters [77]. Reel structure and operating trajectory are also critical to header performance. Using rapeseed as the study crop, Qing et al. reduced reel impact and header loss by optimizing tine trajectories during crop engagement, conveying, and release [78]. This transferable evidence indicates that soybean reel design should consider not only the reel-speed index but also plant branching, lodging severity, and susceptibility to pod shattering to achieve compliant crop lifting and uniform feeding.

Modern large combine harvesters can achieve relatively low losses under favorable crop and field conditions. Regmi et al. reported average loss rates of 0.22–0.57% for several machine models operated at relatively low forward speeds [79]. Nevertheless, the broad variation among studies synthesized by Carreira et al. demonstrates that field conditions, machine condition, parameter configuration, and evaluation methods all substantially affect measured loss [5]. Moreover, soybean loss is commonly distributed non-uniformly across the header width, making single-point sampling unrepresentative of whole-machine performance. Regmi et al. established multiple sampling positions across the working width and found that the locations of peak loss differed among machine models [79]. Loss evaluation should therefore cover the principal header width while simultaneously documenting crop condition, cutting height, reel-speed index, cylinder or rotor speed, concave clearance, and cleaning settings.

Lambrecht et al. further showed that harvest loss may be concentrated near either the center or the ends of the header and that the sampling range and point density should be adapted to header width [80]. The complete working width should be assessed for narrow headers, whereas any reduction in the sampling range for large headers should still retain multiple consecutive sampling positions. A uniform single-point protocol is therefore unsuitable for comparing different machine models. Lodging direction is another spatial factor affecting stable header operation. Using wheat as the study crop, Zhang et al. applied machine vision to identify lodging direction and provide information for travel-path and header-state adjustment [81]. A corresponding soybean system could integrate lodging-direction sensing with travel-direction planning, reel adjustment, and header-height control to reduce crop pushing, missed cutting, and feeding blockage.

Direct combine harvesting must coordinate loss reduction with the preservation of grain quality. Regmi et al. found that machine models with lower harvest losses did not necessarily produce lower grain breakage or seed-coat damage [79]. Increasing threshing intensity or cleaning airflow may reduce unthreshed grain and impurity content but may simultaneously increase mechanical damage or cleaning entrainment loss. Harvest-quality evaluation should therefore include total loss, header loss, grain breakage, seed-coat damage, unthreshed grain, and impurity content.

The process routes, material-state changes, field efficiency, labor requirements, weather dependence, quality-protection capacity, principal risks, control priorities, and applicable conditions of manual cutting and stationary threshing, mechanized windrowing and pickup, and direct combine harvesting are compared, as shown in Table 2. These three harvesting routes do not represent a simple performance ranking; instead, each is suited to different production scales, crop conditions, and quality objectives.

Manual cutting and stationary threshing are highly adaptable to low pod positions and complex field conditions and can effectively preserve seed quality, but they require substantial labor and have limited continuous operating capacity. Mechanized windrowing and pickup regulate crop moisture through field drying and are advantageous for unevenly mature or high-moisture crops, although they introduce risks of windrow shattering, rewetting, and pickup loss. Direct combine harvesting provides the highest field efficiency and is well suited to large-scale production under conditions of uniform maturity, appropriate moisture content, and good field trafficability, but it requires close coordination among header, feeding, threshing, separation, and cleaning settings. Harvesting-method selection should therefore be based on crop maturity, lowest pod height, lodging severity, weather conditions, field scale, and end use rather than on operating efficiency alone. Modern direct combine harvesting can satisfy the efficiency requirements of large-scale soybean production on level fields and maintain low losses when crop conditions and machine settings are properly matched. However, major challenges remain, including aging machinery, non-uniform lateral loss distribution, poor representativeness of single-point sampling, and multi-objective trade-offs between harvest loss and grain quality.

The header remains the primary target for loss reduction in direct combine harvesting. Close-to-ground terrain-following cutting, optimized tine trajectories, and compliant reel conveying can reduce missed low pods and impact-induced grain shedding, while visual sensing can identify lodging direction and crop condition to support dynamic adjustment of the travel path, header height, and reel settings. Future studies should standardize the reporting of crop conditions, machine condition, parameter settings, and sampling positions, as recommended by Carreira et al., to improve cross-study comparability and advance direct combine harvesting from isolated parameter adjustment toward coordinated, whole-machine, multi-objective control [5].

### 3.3. Low-Loss Header Technologies

Research on low-loss soybean headers focuses primarily on missed low pods, adaptation to uneven ground, pod shattering caused by reel impact, and unstable feeding. Although soybean-producing regions differ in terrain, cultivars, and planting systems, headers must simultaneously provide close-to-ground cutting, reliable terrain following, compliant crop gathering, and continuous material conveying. Research has therefore progressed from isolated structural modifications toward analysis of crop–component contact mechanisms, dynamic simulation, and coordinated multi-parameter optimization. Data-driven virtual design has also been introduced to coordinate reel structure and operating parameters in crop-harvesting equipment. Golpira and Sola-Guirado combined field-loss data from five prototype chickpea headers with a fuzzy model and simulated annealing to optimize reel diameter and the number of reel bats, establishing a data-driven method for low-loss design [82]. Although chickpea was used as the target crop, the sequence of prototype testing, data modeling, and virtual optimization provides a useful methodological reference for the coordinated design of soybean reel trajectories, structural dimensions, and operating settings.

For close-to-ground cutting and terrain following, Nie et al. developed a synchronous terrain-following low-loss soybean header incorporating a ground-profile sensing plate, spring mechanism, low-position reciprocating cutterbar, and compliant reel-conveying device [22]. The system adjusted cutterbar height in response to ground undulations and reduced both missed cutting and soil intake relative to a rigid low-cutting header. In field tests, header loss and stubble height were 1.34% and 70.36 mm, respectively, representing reductions of 55.0% and 22.7% compared with the rigid control header. These results demonstrate that synchronous terrain following combined with compliant conveying can improve harvesting quality under low-pod and uneven-ground conditions. On the basis of mechanical terrain following, header-height regulation is developing toward integrated mechatronic–hydraulic control. To address uneven surfaces and low pod position in saline–alkali fields, He et al. designed an adaptive system comprising a mechanical terrain-following mechanism, angle sensor, programmable logic controller, and hydraulic actuator. System response was optimized using a coupled multibody-dynamics–discrete-element model and fuzzy proportional–integral–derivative control [83]. Field tests showed a mean stubble-height deviation below 4 mm, a coefficient of variation below 0.08, and a header-height control error within 10 mm, indicating that the integration of mechanical terrain following with active hydraulic regulation can improve cutting-height consistency under complex surface conditions. For lodged crops, however, reducing header height alone may not be sufficient to lift plants for reliable cutting and feeding. Using lodged ratoon-rice stubble as the study object, Xing et al. developed an eccentric parallel four-bar uprighting device and optimized rotational speed, forward speed, and crop-entry angle through coupled DEM–MBD simulation and response-surface analysis [84]. The optimized device achieved an uprighting rate of 90.35%. Although the crop and operational objective differed from soybean harvesting, the eccentric trajectory, plant-pose regulation, and combined simulation–field-validation approach provide references for soybean crop-lifting mechanisms and reel-trajectory design.

Low-loss gathering also depends on the coordinated matching of reel motion, contact geometry, and material properties. Chen et al. analyzed reel-induced pod shattering from the perspective of tine–pod contact mechanics [85]. The mean rupture force of soybean pods under vertical compression was 14.33 N, while Hertzian contact analysis showed that reducing tine-material elastic modulus and increasing contact area could lower pod contact stress. Optimized polyvinyl chloride–nylon compliant tines reduced maximum equivalent stress by 90.3% in simulation and decreased field header loss to 1.37%. These findings indicate that low-loss gathering is governed not only by reel speed and position but also by tine compliance, contact geometry, and pod mechanical properties. Studies of compliant contact further show that component geometry, contact position, and engagement depth jointly affect damage to biological materials. Han et al. developed a multi-pin compliant seedling gripper using flexible needles and coordinated control for gripping, holding, and release, achieving a maximum seedling-integrity rate of 93.37% [86]. Although designed for transplanting, its multipoint load distribution and compliant elements provide a methodological reference for soybean reel tines, crop dividers, and feeding components. Wang et al. further developed the 4GS-457 low-loss soybean header to coordinate reel trajectory and operating parameters [61]. An eccentric mechanism maintained a relatively stable tine orientation during crop engagement and conveying, thereby reducing impact on pods. Cycloidal kinematic analysis and a Box–Behnken response-surface design were used to optimize reel speed, installation height, and horizontal position. Field validation produced a header loss rate of 1.42%, a stubble height of 100 ± 10 mm, harvest recovery above 98%, and field capacity of 0.69 hm^2^/h, demonstrating the value of integrating structural design with multi-parameter matching for wide-swath and high-efficiency harvesting.

Crop-condition sensing provides an important basis for adaptive header regulation because lowest pod height, lodging severity, and moisture content directly influence the appropriate cutterbar height, reel intensity, and forward speed. Sun et al. used hyperspectral images from 871 to 1766 nm to develop a rice-grain moisture-prediction model. After wavelength selection, the BCC-LS-SVR-SPA model achieved good predictive accuracy, demonstrating the potential of hyperspectral sensing for rapid and non-destructive moisture determination [87]. Although developed for rice-quality assessment, this method provides a reference for online sensing of soybean grain or pod moisture and for coordinated regulation of header and subsequent threshing settings. Overall, low-loss soybean header technology is converging on an integrated pathway comprising close-to-ground terrain-following cutting, lodged-crop lifting, compliant low-impact gathering, continuous conveying, and coordinated parameter optimization. Terrain-following cutting addresses missed low pods and surface undulation; compliant reels and multipoint-contact components reduce pod collision and local stress; crop-lifting mechanisms improve the cutting and feeding of lodged plants; and crop-condition sensing enhances adaptability across cultivars and field conditions. Existing validation nevertheless remains concentrated on specific cultivars, machine models, and field conditions, while uprighting, compliant-gripping, and spectral-sensing methods transferred from other crops have not yet been systematically validated on soybean headers. Future research should therefore conduct multi-region and multi-season testing under different planting systems and crop conditions and integrate visual, spectral, and terrain sensors to develop adaptive headers capable of automatically regulating cutting height, reel speed, tine trajectory, and installation position according to lowest pod height, lodging direction, pod moisture content, and real-time feed rate.

### 3.4. Low-Loss Threshing, Separation, and Cleaning Technologies

**Figure 8:**
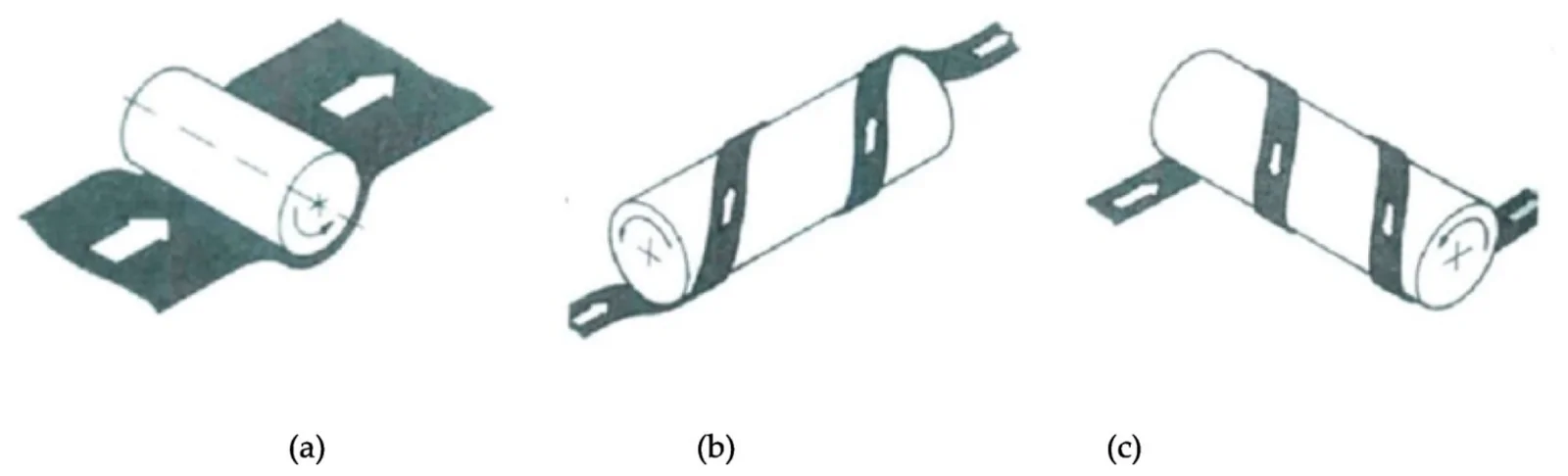
Schematics of three basic threshing-unit configurations illustrated using corn systems: (**a**) tangential-flow, (**b**) transverse axial-flow, and (**c**) longitudinal axial-flow. Reproduced from Li et al. [88], Figure 4, under the Creative Commons Attribution 4.0 International (CC BY 4.0) license. The large arrows indicate the direction of material flow, the curved arrow indicates the rotational direction of the threshing cylinder, the dashed line denotes the cylinder axis, and the cross mark indicates the center of rotation.

Three basic threshing-unit configurations—tangential-flow, transverse axial-flow, and longitudinal axial-flow—are illustrated in Figure 8, using corn-threshing systems as representative structural examples [88].

Threshing, separation, and cleaning are critical to controlling grain breakage, harvest loss, and impurity content during soybean combine harvesting. The threshing unit applies impact, rubbing, and combing actions to open pods and release grain, whereas the cleaning system uses airflow and sieve motion to separate grain from stems, pod walls, leaves, and other non-grain material. Because legume seeds are susceptible to mechanical damage, pods shatter readily, and the composition of the material stream varies substantially, directly applying conventional cereal-harvester settings often results in excessive grain breakage, cleaning loss, and unstable performance [89]. The central objective of low-loss threshing is therefore to balance threshing completeness against grain damage. Increasing cylinder speed or threshing intensity can reduce the unthreshed-grain rate but also intensifies impact, shear, and compression. Ukatu optimized the geometry of soybean threshing teeth to reduce contact impact while maintaining threshing efficiency above 99%, thereby decreasing grain damage from 1.94–2.43% to 0.83–0.98% [90]. Appropriate threshing-element geometry and clearance can therefore improve threshing completeness without causing unacceptable mechanical damage.

Mechanical damage accumulates across multiple machine stages and should not be considered solely within the threshing unit. Ni et al. found that seed-coat injury, cotyledon splitting, and complete grain breakage occurred at the feed auger, threshing cylinder, and grain-conveying system and were strongly influenced by forward speed and cylinder speed [91]. Low-loss design should therefore coordinate rotational speed, collision frequency, drop height, and material-flow stability throughout feeding, threshing, separation, and conveying. The threshing and separation configuration also affects grain breakage and downstream cleaning load. Jasper et al. compared single-rotor and twin-rotor axial-flow combines and found that the twin-rotor machine produced lower grain breakage, impurity content, and unthreshed-grain rates [92]. Its larger effective separation area and clearance promoted rapid grain passage and reduced repeated impacts, indicating that low-damage threshing can also be achieved by increasing separation capacity and shortening material residence time. Using flax as the study crop, Shi et al. applied coupled discrete-element and finite-element analysis to optimize threshing-cylinder structure and found that a combination of rasp bars and spike teeth improved material motion and separation while reducing mechanical load [93]. This simulation approach provides a useful reference for the combined design and virtual evaluation of soybean threshing elements.

With advances in sensing and control, threshing adjustment is shifting from fixed settings toward condition-adaptive regulation. Liu et al. estimated feed rate from header height, grain moisture content, and feeder-house image features and adjusted the clearances of the threshing and separation sections in real time [14]. This study illustrates the transition from fixed threshing settings toward condition-adaptive regulation based on material-state sensing. Feed rate, moisture content, and material-flow condition are therefore essential inputs for dynamic matching of threshing parameters. During cleaning, fan speed, air-inlet opening, sieve opening, cleaning-shoe motion, and feed load jointly determine cleaning loss and impurity content, while the proportions of grain, stem fragments, pod walls, and leaves in the threshed mixture vary with cultivar, maturity, moisture content, and operating load. Fixed settings cannot therefore maintain stable performance during prolonged operation under changing crop conditions. Liang and Wada emphasized that cleaning performance depends not only on individual fan and sieve settings but also on airflow distribution, the feeding state of the threshed mixture, cleaning-shoe motion, and material-layer thickness [36]. Soybean cleaning systems should consequently be optimized as coupled structure–material–parameter systems rather than through isolated increases in airflow or sieve opening.

Liu et al. developed an online system for monitoring soybean cleaning loss and impurity content and coordinating fan speed, air-inlet opening, sieve-louver opening, and cleaning-shoe crank speed [8]. The study demonstrates that quality feedback and coordinated multi-parameter regulation can improve the adaptability of soybean cleaning systems under variable operating conditions. Using rice as the study material, Ding et al. combined terminal-velocity testing with coupled DEM–CFD analysis to optimize a multi-duct cleaning structure and substantially reduce sieve loss and impurity content [94]. This approach demonstrates that airflow should be organized according to aerodynamic differences among grain, stem fragments, and light impurities and provides a reference for gas–solid coupling analysis and air-duct design for soybean-threshed mixtures. Overall, soybean threshing, separation, and cleaning technologies are progressing toward low-impact threshing, high-efficiency separation, continuously adjustable clearances, condition monitoring, optimized airflow distribution, and adaptive multi-parameter control. Threshing intensity should be regulated according to grain moisture content, pod condition, and feed rate, whereas air-and-sieve settings should be matched dynamically to material composition, layer thickness, and operating load. Future research should strengthen coordinated optimization of threshing and cleaning and use discrete-element, computational-fluid-dynamics, and multibody-dynamics models to clarify the continuous interactions among pod opening, grain release, material dispersion, and aerodynamic separation. Parameter databases should cover cultivar, maturity, moisture content, straw-to-grain ratio, and feed rate, while grain breakage, unthreshed grain, threshing and separation loss, cleaning loss, and impurity content should be incorporated into multi-objective control. The long-duration reliability of intelligent regulation should also be validated across machine models, regions, and complex operating conditions.

### 3.5. Applicable Scenarios and Technical Boundaries of Harvesting Methods and Equipment Configurations

Soybean-harvesting methods differ in field efficiency, loss-control potential, equipment investment, and environmental adaptability. Direct combine harvesting provides the highest field efficiency, but its performance depends jointly on terrain, crop condition, header configuration, and operating-parameter matching. Menezes et al. compared flex and auger headers and found that the flex header maintained a lower and more stable stubble height and reduced header loss by 35–51%, demonstrating that improved ground conformity can reduce missed low pods [77]. However, header loss still accounted for 70–80% of total loss in those tests, indicating that header replacement alone cannot achieve comprehensive loss control; crop condition, machine settings, and operational management must also be coordinated. Nie et al. showed that a synchronous terrain-following soybean header integrating close-to-ground cutting, compliant gathering, and stable conveying can extend the applicability of direct combine harvesting under low-pod and uneven-ground conditions [22]. Two-stage harvesting, by contrast, regulates material condition through cutting and windrowing, field drying, pickup feeding, and subsequent threshing and cleaning. It is therefore more suitable for high-moisture, lodged, or unevenly mature crops, although it requires additional operations and specialized equipment. Direct combine harvesting remains generally preferable for large, level fields with uniform maturity and suitable grain moisture content.

Planting systems, field scale, terrain, and mechanization levels vary substantially among regions, and no single machine configuration can accommodate all production scenarios. In a review of potato harvesting, Song et al. noted that regional heterogeneity and insufficient equipment adaptability restrict machinery adoption [95]. Their approach of combining differentiated machine configurations with multiple operating modes provides a useful reference for matching soybean-harvesting equipment to regional production conditions. Field geometry and uninterrupted operating distance also influence combine-harvesting efficiency. Paixão et al. found that operational efficiency was substantially higher in rectangular fields than in irregular or trapezoidal fields [96]. The efficiency advantage of large combines therefore depends not only on field area but also on regular field boundaries, fewer headland turns, and adequate continuous travel distance. Accordingly, large-scale soybean production on level and regular fields is best served by combine harvesters equipped with flexible or crop-specific low-loss headers, whereas crops with low pods, severe lodging, uneven maturity, or complex terrain may require specialized low-loss equipment or two-stage harvesting. Small plots, seed production, and quality-sensitive end uses may be better served by compact machines, mechanized windrowing, and compliant threshing systems.

Equipment matching for high-quality soybean should extend beyond harvesting components to include grain unloading and transfer. Albaneze et al. found that auger-based unloading and transfer increased soybean grain breakage and seed-coat damage, particularly at low seed moisture contents [97]. Seed soybean and other quality-sensitive products should therefore minimize the number of transfer operations and adopt low-impact conveying and buffered handling. Internal combine configuration also involves trade-offs among energy consumption, grain quality, and operating cost. Jasper et al. found that single-rotor machines had higher energy efficiency, whereas twin-rotor machines produced lower grain breakage, impurity content, and unthreshed-grain rates [92]. Machines with larger separation areas and shorter material residence times may therefore be more suitable for quality-sensitive soybean, while single-rotor systems may be preferable when energy consumption and cost are the dominant constraints. Even within a given harvesting route, the relationship between forward speed and grain quality is not simply linear. High-quality harvesting should therefore rely on coordinated multi-parameter regulation rather than on reducing forward speed in isolation.

Visual sensing and online quality monitoring can further improve equipment adaptability and support real-time parameter adjustment. In a review of selective fruit-harvesting robots, Wang et al. summarized methods for target recognition and environmental perception [98]. These methods provide references for detecting soybean-field boundaries, lodged zones, crop strips, and obstacles and for supporting local path planning and operating-parameter regulation. Online loss monitoring can also provide real-time feedback for machine adjustment. Using wheat as the study crop, Shen et al. estimated grain loss from the acoustic-impact characteristics of grain and stems and achieved a mean field-detection error of 6.1% [99]. This method provides a transferable reference for monitoring soybean header, threshing, separation, and cleaning losses, although its robustness to cultivar, moisture content, machine vibration, and background noise requires further improvement. Future research should establish quantitative matching models linking harvesting routes and machine settings with terrain, field scale, planting system, cultivar traits, grain moisture content, and quality objectives. Visual sensing, online loss monitoring, and quality-feedback control should be integrated to optimize field efficiency, harvest loss, grain quality, energy consumption, and operating cost within a unified decision framework, while unloading, transfer, and post-threshing handling should also be incorporated to prevent additional quality deterioration.

## 4. Intelligent Sensing and Adaptive Control Technologies

This section is organized around a closed-loop sensing–decision–actuation–feedback chain for intelligent soybean harvesting. Section 4.1 and Section 4.2 address sensing and state estimation, Section 4.3 addresses navigation and motion decision-making, and Section 4.4 focuses on actuator regulation and harvest-quality feedback.

Within this chain, the reviewed intelligent methods can be grouped into three functional layers. Perception-oriented AI converts raw visual, point-cloud, impact, acoustic, vibration, and spectral data into observable crop, terrain, material-flow, and harvest-quality information through image processing, semantic segmentation, convolutional neural networks, Transformer-based models, and point-cloud learning. State-estimation and prediction methods, including regression, support-vector machines, neural networks, and multi-source fusion, map heterogeneous sensor signals to variables such as feed rate, loss state, moisture condition, and blockage risk. Decision and control intelligence then uses fuzzy logic, predictive control, data-driven system identification, and multi-objective optimization to translate estimated states and quality feedback into actuator commands. This functional classification emphasizes that AI is not an isolated algorithmic layer but a set of methods embedded throughout the sensing–decision–actuation–feedback loop.

### 4.1. Monitoring of Harvest Loss, Grain Breakage, Impurity Content, and Feed Rate

Online monitoring of harvest quality is fundamental to the intelligent control of soybean combine harvesters. Liu et al. installed piezoelectric sensors at the rear sieve-discharge outlet and clean-grain outlet and estimated cleaning loss and impurity content by analyzing grain-impact signals [8]. These measurements provide real-time quality feedback for subsequent cleaning-parameter regulation. Further optimization of sensing-plate structures and vibration features can improve the recognition of grain-impact signals under strong machine vibration and provide a basis for interference-resistant online monitoring of soybean harvest loss [100]. Machine vision also enables online monitoring of grain breakage through controlled sampling and image classification. Using rice as the study material, Chen et al. developed a grain-tank sampling and imaging device and identified broken grains from color features with a recognition accuracy of 96% [101]. The workflow of representative sampling, stable illumination, controlled imaging, and damage classification provides a useful reference for the online estimation of soybean grain breakage. Impurity monitoring must additionally address material occlusion, sampling representativeness, and the conversion of image features into mass-based impurity content. Using rice as the study material, Guan et al. combined a conveying and sampling device, discrete-element analysis, and Mask R-CNN to identify impurities in a grain stream, validating a workflow comprising stable sampling, controlled illumination, object segmentation, and pixel-to-mass calibration [102]. Application to soybean requires model retraining and calibration for grains, pod husks, stem fragments, and leaves. For soybean-threshed mixtures, an edge-sensing CNN–Transformer architecture can classify impact features from different material components and provides an algorithmic basis for edge-side estimation of impurity content and loss state [103].

Feed rate directly determines threshing and separation load and strongly affects harvest quality. Liu et al. combined feeder-house image transmittance, header height, and grain moisture content in a particle-swarm-optimized backpropagation neural network to predict feed rate, achieving a mean field-monitoring accuracy of 90.8% [14]. This result demonstrates that multi-source sensing can effectively represent dynamic variations in material flow. Feed rate can also be inferred indirectly from mechanical load. Jiang et al. measured torque on the header-auger input shaft and used a support vector machine to establish a torque–feed-rate relationship with a coefficient of determination of 0.967 [104]. Torque sensing can complement image, header-attitude, and moisture measurements and improve feed-rate estimation when dust, occlusion, or variable illumination reduces the reliability of visual sensing. Using a vibratory grain-feeding process, Zhang et al. established a regression relationship between near-infrared reflectance features and material flow rate, enabling non-contact online measurement [105]. This approach provides an additional pathway for estimating grain velocity and mass flow in the feeder house or clean-grain elevator of a soybean combine harvester. Because rapid feed-rate fluctuations can cause abrupt changes in threshing-cylinder load and lead to blockage, Zhu et al. fused acoustic and vibration signals, extracted time- and frequency-domain features, and identified operating states and blockage trends in a threshing and separation unit [106]. This method provides a reference for soybean threshing-load monitoring, early blockage warning, and adaptive parameter regulation.

**Figure 9:**
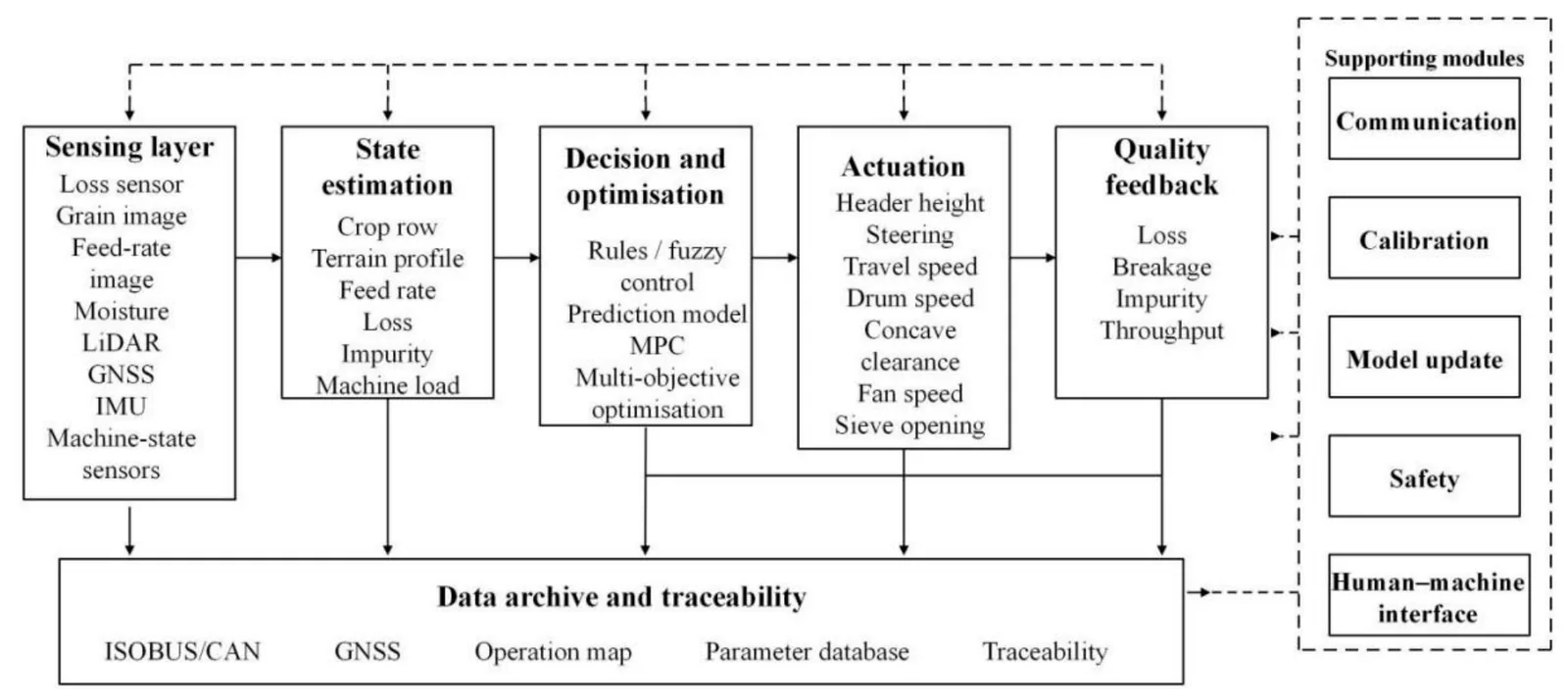
Integrated sensing–decision–actuation–feedback architecture and operating-data chain for intelligent soybean combine harvesting. Solid arrows indicate the main information and control flow among sensing, state estimation, decision and optimisation, actuation, quality feedback, and data archiving, whereas dashed arrows indicate auxiliary communication and supporting links.

The ultimate purpose of online sensing is to support closed-loop regulation through coordinated sensing, decision-making, actuation, and feedback. Zhang et al. tested a single-chip-microcomputer and fuzzy-control system for regulating fertilizer concentration in fertigation equipment [107]. This provides an architectural analogy for connecting sensing, control decisions, and actuators, but it does not validate soybean-harvester quality feedback, response delay, or field robustness. These monitored variables provide the feedback signals required for closed-loop regulation of cleaning and threshing parameters [8,14]. The soybean-specific studies cited above [8,14] provide the harvest-related basis for converting monitored variables into control objectives; the fertigation study [107] supports only the general control architecture. The integrated sensing–decision–actuation–feedback architecture and operating-data chain for intelligent soybean combine harvesting are shown in Figure 9.

Existing research has thus established an initial technical chain integrating multi-source sensing, state estimation, parameter decision-making, actuator regulation, and quality feedback. Online monitoring of cleaning quality and feed rate reduces dependence on operator experience and provides the data required for coordinated threshing-cleaning control and higher-level whole-machine decision-making.

### 4.2. Sensing of Crop, Terrain, and Machine States

Sensing of crop, terrain, and machine states provides the information required for autonomous navigation, header terrain following, and adaptive parameter regulation. Crop-row position, canopy structure, and plant distribution affect row-following accuracy and feeding uniformity, whereas ground undulations, header height, and actuator state determine stubble-height consistency, low-pod recovery, and the risk of cutterbar–ground contact. Mature soybean canopies are often closed and affected by occlusion, missing plants, lodging, and weeds, driving a transition from two-dimensional vision toward LiDAR and three-dimensional point-cloud sensing. Multi-source UAV imagery combined with self-supervised contrastive learning has been used to estimate soybean yield and classify lodging severity, providing complementary information for preharvest prescription mapping and the identification of high-risk areas within fields [108]. Using maize at the middle and late growth stages as the study crop, Zhang et al. applied solid-state LiDAR to extract crop-row centerlines under closed-canopy conditions [109]. The results demonstrate the value of three-dimensional geometric information when inter-row boundaries are indistinct and provide a reference for crop-row detection in mature soybean.

LiDAR-based mapping and plant-structure recognition can further support autonomous navigation and crop-state assessment in complex harvesting environments. Chen et al. fused LiDAR and inertial-measurement-unit data to construct an orchard point-cloud map and complete inter-row and headland path planning with low lateral navigation error [110]. Their workflow of point-cloud mapping, structural-feature extraction, and path control provides a reference for autonomous navigation of soybean combine harvesters under unstable GNSS reception or complex crop-strip boundaries. To address overlapping soybean canopies, branch and leaf occlusion, and variations in planting density, Fang et al. used three-dimensional LiDAR to extract point clouds near the bases of main stems and determine crop-row centers [111]. Dynamic grid division, outlier removal, and interpolation across missing-plant areas achieved a mean detection accuracy of 90.6%, a mean angular error of 2.35° ± 0.59°, and a single-frame processing time of approximately 122.7 ms, satisfying real-time navigation requirements. Using nursery trees as the study objects, Xu et al. applied D-PointNet++ to classify and segment point clouds of individual plant components [112]. This method provides a transferable reference for distinguishing soybean main stems, canopies, lodged areas, and weeds and could improve crop-row detection, feed-state estimation, and obstacle recognition.

Terrain and header-state sensing are equally important for maintaining stable close-to-ground cutting. Li et al. used area-array LiDAR to measure ground elevation ahead of the machine and reconstructed the terrain through outlier removal and robust fitting [113]. Compared with contact-based terrain following, non-contact sensing can detect surface changes in advance, support predictive header-height control, and reduce wear and false responses in mechanical sensing elements. Header height must also be estimated reliably under vibration, stubble interference, and weed disturbance. Wang et al. fused information from a contact terrain-following mechanism with angle, position, and velocity measurements and used Kalman prediction and fuzzy decision-making to suppress disturbances [114]. In field tests, most height-estimation errors were within −5 to 5 mm, indicating that multi-source fusion can improve the stability of header-state estimation. Liu et al. further developed a terrain-following mechanism capable of adjusting both header height and lateral attitude and analyzed terrain–mechanism–crop interactions using a coupled multibody-dynamics/discrete-element model [115]. Their results show that terrain-following control should integrate terrain profile, header attitude, mechanism motion, and ground-contact state rather than rely on a single height measurement.

Hydraulic-actuator lag remains a major constraint on rapid terrain following. Xie et al. noted that header-height systems are limited by the spatial separation between sensing and actuation and by restricted hydraulic bandwidth [116]. As forward speed increases, these limitations may cause control delay and height overshoot, requiring feedforward or lead compensation based on terrain detected ahead of the header. Li et al. used a header-lift model and position-threshold lead compensation to actuate the hydraulic system in advance and achieved a field-control error of approximately 5 mm, lower than that obtained under manual control [113]. Zhang et al. further limited the mean stubble-height deviation to within 4 mm, demonstrating that predictive control can improve cutting-height stability on undulating terrain [117]. Overall, sensing for soybean harvesting is progressing from isolated indicator measurement toward multi-source fusion of crop-row recognition, plant-structure segmentation, terrain reconstruction, machine-state estimation, and actuator feedback. LiDAR and point-cloud algorithms can identify crop rows, canopies, lodged areas, and ground structure, whereas angle, displacement, pressure, and hydraulic sensors can estimate header attitude and actuator state. Future research should strengthen the fusion of LiDAR, machine vision, satellite positioning, inertial measurement, and machine-mounted sensors to improve reliability under dust, vibration, intense illumination, lodging, and dense-canopy conditions. Header height, travel direction, forward speed, and feed rate should be regulated coordinately according to crop-row position, terrain ahead of the machine, header attitude, and hydraulic-response state.

Although vision and LiDAR have shown promising results for crop-state sensing, the reviewed soybean studies do not consistently quantify how accuracy changes with dust, vibration, illumination variation, severe lodging, missing plants, or dense-canopy occlusion. The mature-soybean LiDAR study specifically considered planting density, weed interference, and missing-plant gaps [111], but its aggregate performance results do not provide controlled degradation curves for all of these disturbances. Missing quantitative data should therefore be identified as a validation gap rather than converted into assumed percentage losses. Future evaluations should report crop-row detection accuracy, angular error, false detections, sensor dropout, and recovery time for defined disturbance levels while holding crop state and machine speed comparable. Matched camera-only, LiDAR-only, and fused-sensing tests would further establish whether sensor redundancy improves perception continuity when individual measurements become unreliable.

### 4.3. Header Terrain Following, Vehicle Navigation, and Path Control

Header terrain following, vehicle navigation, and path control directly affect stubble-height consistency, effective cutting width, and feeding stability. Mature soybean fields may contain closed canopies, missing plants, lodging, weeds, and indistinct crop-strip boundaries, making continuous operation based on a single two-dimensional vision sensor unreliable. Recent research has therefore increasingly combined three-dimensional LiDAR, binocular vision, and multi-sensor fusion for crop-row recognition, navigation-line generation, harvest-boundary detection, and forward terrain sensing. Ma et al. noted that crop-row detection is affected by growth stage, planting density, illumination, weed pressure, and lodging severity [7]. Navigation systems should therefore be evaluated not only in terms of recognition accuracy but also according to processing speed, temporal continuity, and robustness under complex field conditions. Vision-based, LiDAR-based, and fused sensing configurations should be selected according to soybean canopy closure and operating conditions. To address canopy occlusion and variations in planting density, Fang et al. used three-dimensional LiDAR to extract point clouds near the bases of main stems and determine crop-row centers [111]. Dynamic grid division removed false rows caused by weeds, while interpolation restored continuity across missing-plant areas. The method achieved an overall detection accuracy of 90.6% and a mean crop-row angular error of 2.35° ± 0.59°, providing a basis for real-time row-following navigation.

Crop-row centerlines must subsequently be transformed into navigation references that can be used by the vehicle controller. In a soybean–maize strip-intercropping environment, Ou et al. used point-cloud clustering and centerline fitting to generate a travel line with a mean angular deviation of 0.28° [118]. This study demonstrates that LiDAR output can be converted into heading error and lateral offset for path tracking under strip-planting conditions, although performance still requires validation during the actual harvesting of mature soybean. Navigation based solely on crop-row centerlines may be interrupted by missing plants, lodging, or indistinct row traces. Using rice, rapeseed, and maize as study crops, Luo et al. applied binocular vision to identify unharvested crop edges and headland boundaries and verified their value for straight-line travel and headland row changes [119]. Combining crop-row tracking with supplementary harvest-boundary detection can therefore improve path continuity and headland turning in soybean fields.

The core task of header terrain following is to detect ground undulations in advance and convert the reconstructed terrain profile into header-height commands. Li et al. used area-array LiDAR to measure ground elevation ahead of the header, extracted a reference terrain profile through outlier removal and robust fitting, and implemented lead compensation based on a header-lift model [113]. The absolute stubble-height error under automatic control was approximately 5 mm, compared with 8 mm under manual control, showing that forward terrain sensing can reduce height fluctuations caused by hydraulic lag. Zhang et al. further combined area-array LiDAR with a Bang–Bang predictive switching strategy, enabling the solenoid valve to actuate before the height error was fully developed [117]. Simulation and bench tests demonstrated improved terrain tracking and disturbance rejection; however, the long-duration stability of this approach under soybean field conditions still requires systematic validation. Header terrain following and vehicle control should therefore form a coordinated chain comprising crop-row and harvest-boundary recognition, navigation-line generation, path tracking, forward terrain sensing, and predictive header-height regulation. The controller should also account for forward speed, header vibration, and hydraulic-response lag to reduce missed low pods and cutterbar–ground contact while maintaining effective cutting width and uniform feeding.

**Figure 10:**
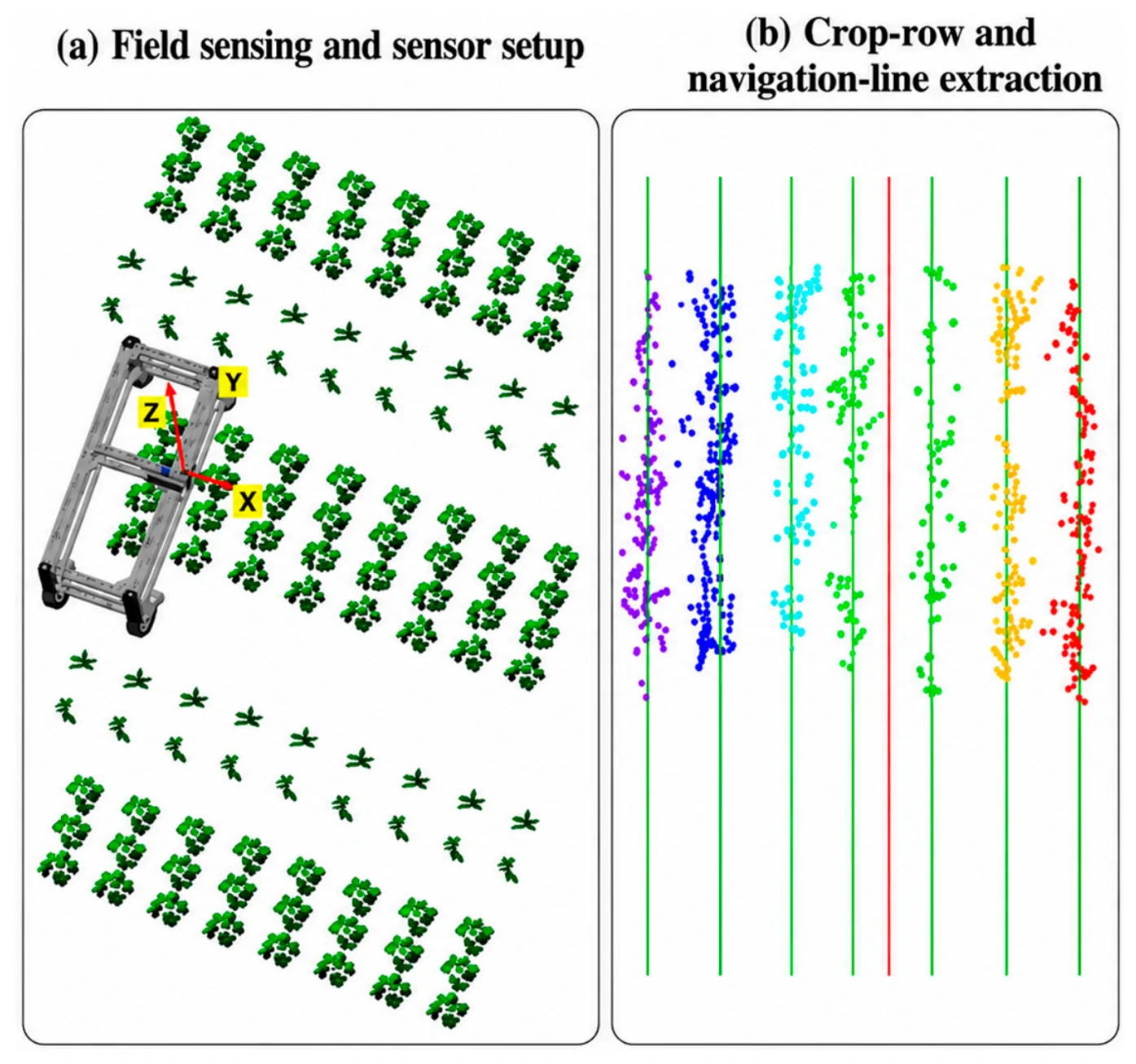
LiDAR-based field sensing, crop-row extraction, and navigation-line generation. (**a**) Schematic of field crop point-cloud acquisition and LiDAR sensor arrangement. (**b**) Crop-row clustering and navigation-line extraction; different point colors distinguish separate clustered crop-row point sets for visualization, the green lines indicate the fitted crop-row centerlines, and the red line indicates the extracted navigation line. Panel (**a**) was adapted from Figure 1 of Ou et al. [118], and panel (**b**) was adapted from Figure 9 of Ou et al. [118], under the Creative Commons Attribution 4.0 International (CC BY 4.0) license.

To connect field sensing with navigation and header control, raw LiDAR measurements must be converted into control-oriented state variables, as illustrated in Figure 10. Point-cloud processing removes ground and noise interference; identifies stem or basal points; and extracts crop-row centerlines, harvest boundaries, and navigation lines. Heading angle, lateral offset, forward terrain profile, and target header height then become the principal inputs for steering and cutting-height control. These variables can be fused with GNSS, IMU, and machine-state information and transmitted to the controller, which generates commands for steering and hydraulic header-lift actuation. Feedback on the actual travel path, harvest boundary, header position, and stubble height closes the loop. Figure 10 therefore retains only (a) field crop point-cloud acquisition and LiDAR sensor arrangement and (b) fitted crop-row centerlines and the extracted navigation line; detailed controller-electronics circuitry is not included, because it is not essential to the review discussion.

At the whole-machine level, local crop-row navigation must be integrated with global position, heading, coverage-path planning, and headland-turning information provided by GNSS. Lee et al. developed a dual-GPS-antenna autonomous navigation system incorporating an alpha-shaped path-generation and tracking algorithm for a crawler combine harvester [120]. During rice harvesting, the mean path errors were 0.038 m during straight travel and 0.195 m during headland turns, whereas the corresponding errors without a harvesting load were 0.052 and 0.207 m. These results demonstrate that global positioning, heading estimation, coverage-path generation, and turning control can form a complete autonomous-driving chain for combine harvesters. A review of path planning and tracking in complex orchard environments further showed that global planning primarily addresses field coverage and overall route efficiency, whereas local planning responds to dynamic obstacles, narrow passages, and temporary path changes. Path tracking must additionally account for vehicle kinematics, tire or track adhesion, actuator lag, and speed variation [121]. Autonomous soybean harvesting should therefore combine global coverage planning, local obstacle avoidance, and vehicle path tracking rather than rely solely on a predefined navigation line. Headland turns, irregular field boundaries, transport vehicles, and localized lodging may all require online replanning. Fields2Cover provides modular functions for headland generation, swath generation, route planning, and trajectory generation and can support standardized comparison of coverage-planning algorithms in irregular soybean fields [122].

Soybean-harvester navigation and header terrain following are consequently developing toward an integrated system that combines crop-row and harvest-boundary recognition, terrain reconstruction, navigation-line generation, global and local path planning, machine-state estimation, and actuator feedback. Vision and LiDAR provide crop and terrain information; GNSS and IMU provide global position and attitude; and displacement, angle, pressure, and hydraulic sensors characterize header and actuator states. Future systems should further integrate LiDAR, binocular vision, satellite positioning, inertial measurement, and machine-operation sensors to improve reliability under dust, vibration, intense illumination, lodging, missing plants, and dense-canopy conditions. Environmental perception, global path planning, local obstacle avoidance, path tracking, and header control should be coordinated, while travel direction, cutting height, forward speed, and feed rate should be adjusted dynamically according to crop-row position, harvest boundaries, forward terrain, and real-time operating load.

### 4.4. Quality-Feedback-Based Closed-Loop Regulation of Threshing and Cleaning Parameters

Threshing and cleaning directly determine soybean grain breakage, harvest loss, and impurity content. Conventional operation relies largely on operator experience to set concave clearance, airflow, and sieve parameters and cannot respond promptly to changes in feed rate, moisture content, and threshed-mixture composition. Advances in sensing, intelligent actuation, and control algorithms are therefore shifting these systems from fixed settings toward state-aware decision-making and quality-feedback control. For concave-clearance regulation, Liu et al. used the estimated feed rate to adjust the clearances of the threshing and separation sections according to material load [14]. The regulation reduced grain breakage from 3.39% to 2.36% and harvest loss from 1.87% to 0.70%. Feed-rate monitoring based on reel force can further integrate forward speed, reel speed, header height, and plant-bending force to provide a low-latency feedforward signal for threshing and cleaning control [123]. Because model-based feedforward regulation cannot fully eliminate deviations caused by material fluctuations and prediction errors, real-time quality feedback is also required. Using maize as the study crop, Yu et al. combined an entrainment-loss sensor with fuzzy neural-network PID control to adjust concave clearance and substantially reduce entrainment loss [124], demonstrating that the integration of feed-load prediction and loss feedback can improve control robustness.

For cleaning control, Liu et al. used fuzzy logic to coordinate sieve opening, air-gate opening, fan speed, and cleaning-shoe crank speed according to measured cleaning loss and impurity content [8]. Field tests reduced these two indicators from 0.38% and 2.66% to 0.19% and 1.68%, respectively, demonstrating that coordinated multi-parameter regulation driven by quality feedback can improve cleaning stability. However, the cleaning process is nonlinear, time-varying, and affected by material-transport and actuator delays, meaning that adjustment based only on the current loss value may respond too late. Using rapeseed as the study crop, Chai et al. combined grey prediction with fuzzy control and adjusted sieve opening in advance according to the predicted loss trend [125]. This method provides a reference for delay compensation and predictive regulation in soybean cleaning systems. Threshing and cleaning are also strongly coupled: concave clearance affects grain release, breakage, and entrainment loss and alters the composition and loading of material entering the cleaning shoe, whereas airflow and sieve settings subsequently determine cleaning loss, impurity content, and throughput. The combined process should therefore be regarded as a nonlinear, time-delayed, multi-input–multi-output system with substantial interactions among variables. Li et al. used threshing clearance, sieve opening, and fan-inlet area as inputs and grain breakage, impurity content, and threshing and cleaning losses as outputs and applied a particle-swarm-optimized wavelet neural network for system identification [32]. The model described the coupled relationships between operating parameters and harvest quality, and it provides a basis for coordinated controller design, although its closed-loop performance still requires validation under soybean field-harvesting conditions.

To clarify the nonlinear multi-input–multi-output (MIMO) relationships among operating parameters, crop conditions, and harvest-quality indicators, the coupled threshing–cleaning process can be conceptually expressed as

$$y_{k}=F\left(u_{k-\tau_{u}},d_{k-\tau_{d}}\right)+\varepsilon_{k}$$

where

$$u_{k}={\left[c_{k},n_{t,k},n_{f,k},s_{k}\right]}^{T},d_{k}={\left[q_{k},M_{k}\right]}^{T},$$

and

$$y_{k}={\left[B_{k},L_{t,k},L_{c,k},I_{k}\right]}^{T}.$$

Here, u_k_ denotes the control-input vector at discrete time step, k, where c_k_ is the threshing concave clearance, n_t,k_ is the threshing drum rotational speed, n_f,k_ is the cleaning fan speed, and s_k_ is the sieve opening. The vector d_k_ represents the operating-condition variables, in which q_k_ denotes the crop feed rate and M_k_ denotes the material moisture content. The output vector, y_k_, contains the principal harvest-quality indicators, where B_k_ is the grain breakage rate, L_t,k_ is the threshing loss rate, L_c,k_ is the cleaning loss rate, and I_k_ is the impurity rate. The superscript T denotes the transpose operation.

The nonlinear mapping, ℱ(⋅), represents the coupled relationships among machine-control parameters, operating conditions, and harvest-quality responses. The terms τ_u_ and τ_d_ denote the effective delays associated with control actions and variations in operating conditions, respectively, while ε_k_ represents unmodeled dynamics, external disturbances, and measurement uncertainty. This formulation is intended as a conceptual representation of the nonlinear MIMO characteristics of the threshing–cleaning system rather than as an experimentally calibrated soybean-harvesting model. In practical applications, the nonlinear relationships, interaction strengths, and effective delays must be identified and validated under representative field conditions before the model can be used for model-based predictive or coordinated adaptive control. Nevertheless, the formulation provides a useful theoretical framework for describing the interactions among multiple actuators, changing crop states, and competing harvest-quality objectives.

Existing intelligent regulation systems generally adopt an architecture integrating state sensing, decision-making, actuator control, and quality feedback. The sensing layer acquires feed rate, moisture content, harvest loss, grain breakage, and impurity data; the decision layer determines parameter corrections using parameter-matching models, neural networks, predictive algorithms, or fuzzy rules; and the actuation layer adjusts clearances, rotational speeds, and openings through electric, hydraulic, or electrohydraulic devices. Sun et al. compensated for unknown disturbances in agricultural-vehicle control through disturbance observation and robust control [126]. Similar methods could be used to correct soybean threshing and cleaning settings online when sudden changes in feed rate, moisture content, or mixture composition cause the operating state to deviate from the nominal model. Future systems should therefore use feed rate, moisture content, and material composition as feedforward inputs and grain breakage, threshing loss, cleaning loss, and impurity content as feedback constraints. Threshing and separation clearances, fan speed, sieve opening, and cleaning-shoe motion should be optimized coordinately, while data-driven system identification, online model updating, disturbance observation, predictive control, and multi-objective decision-making should be integrated into a closed-loop system linking material-state sensing, coordinated threshing–cleaning control, multi-actuator execution, and harvest-quality feedback.

From a control perspective, this architecture is best interpreted as a coordinated feedforward–feedback system. Feed rate, moisture content, crop condition, and threshed-mixture composition provide feedforward state information for anticipating changes in machine load, whereas grain breakage, threshing loss, cleaning loss, and impurity content provide feedback constraints on realized harvest quality. These information streams should be mapped to coordinated commands for concave clearance, cylinder or rotor speed, fan speed, sieve opening, cleaning-shoe motion, forward speed, and other available actuators. Such sensor-to-decision-to-actuator coupling is essential for moving from parameter recommendation toward genuine closed-loop adaptive harvesting.

Control-system delay should be separated from algorithm execution time. For terrain following, sensing look-ahead distance and forward speed determine the time available before a detected ground change reaches the cutterbar, while hydraulic dynamics limit the achievable header response [113,116,117]. For threshing and cleaning, measured grain loss or impurity is additionally delayed by crop transport and residence time, so a controller reacting only to the latest outlet measurement may act after the corresponding disturbance has passed [8,125]. Practical control design should therefore quantify sensor and communication latency, material-transport delay, actuator rise and settling times, and their variation with feed rate. Feedforward load estimation, predictive delay compensation, and rate-limited feedback should be tested with synchronized input, actuator, and quality measurements. The available soybean quality-control studies demonstrate improvements under the tested conditions but do not establish whole-machine delay margins or closed-loop stability over rapidly changing field loads.

To compare the reported evidence more systematically, Table 3 summarizes representative soybean-specific and transferable sensing, monitoring, and adaptive-control studies in terms of crop, method, target variables, experimental conditions, reported performance, evidence type, and principal limitations. Adjacent-legume and cross-crop results indicate potentially transferable engineering methods rather than soybean-validated performance; different tasks and test protocols also preclude direct cross-study performance ranking.

Quantitative comparisons indicate that intelligent sensing and adaptive control technologies can achieve measurable improvements in soybean-harvesting quality; however, their effectiveness varies with field conditions, machine configuration, and control objectives. For soybean harvesting, closed-loop parameter regulation based on feed rate and quality monitoring has been reported to reduce grain breakage from 3.39% to 2.36% and harvest loss from 1.87% to 0.70%. Similarly, adaptive cleaning control decreased cleaning-related loss from 0.38% to 0.19% and impurity content from 2.66% to 1.68%. These results demonstrate that sensing-guided adjustment can improve both harvesting efficiency and product quality; however, such improvements are generally obtained under specific experimental conditions and should not be interpreted as universal performance gains for all soybean production systems.

Table 3 also illustrates why numerical sensing results must be interpreted within their validation conditions. Jin et al. [13] reported class-specific F1 scores for soybean-grain images acquired under controlled imaging, whereas Fang et al. [111] reported crop-row accuracy and single-frame point-cloud processing time in mature soybean fields. These are different tasks and performance measures; neither metric alone establishes real-time sensor-to-actuator control performance. In addition, evaluations over a limited set of planting conditions cannot be used to estimate unreported accuracy degradation under dust, vibration, changing illumination, or lodging. The reviewed studies do not consistently provide matched test conditions, processing hardware, or disturbance-stratified timing and accuracy data. Therefore, general claims that machine vision or LiDAR is universally faster or more robust would be premature; comparable field protocols should instead evaluate sensing accuracy, computational time, dropout frequency, and the resulting control quality together.

Despite these advances, several limitations remain regarding technology transfer and field validation. Evidence from adjacent legumes and other crops provides valuable references for sensor development, mechanical design, and control strategies because of shared characteristics such as material flow variation and harvesting damage mechanisms. However, differences in plant architecture, pod distribution, seed mechanical properties, and harvesting operations limit direct transfer of quantitative results across crop species. Furthermore, many existing studies were conducted in laboratory environments, small-scale trials, or limited geographical regions, resulting in insufficient validation under diverse cultivars, terrains, weather conditions, and commercial harvesting scenarios. Future research should therefore focus on large-scale field experiments, standardized evaluation protocols, and long-term validation to confirm the robustness and scalability of intelligent soybean-harvesting systems.

### 4.5. Development of Operating-Parameter Databases and Harvest-Quality Traceability

Operating-parameter databases and harvest-quality traceability systems integrate machine settings, agronomic information, spatial position, yield data, and quality measurements, thereby providing a data foundation for precision-management decisions, equipment optimization, process reconstruction, and long-term evaluation of harvesting performance. Kortenbruck et al. used ISO 11783 (ISOBUS) communication data to collect engine torque, rotational speed, rear-hitch position, wheel speed, and other signals through a CAN-bus data logger and fused these data with GNSS information to identify field boundaries and classify operating states [127]. Primary operation accounted for 56.4% of field time and headland turning for 23.7%, demonstrating the feasibility of automatically archiving machinery operating states from communication-bus data. Wang et al. subsequently developed the ISOBlue HD platform to synchronously record CAN-bus, GNSS, camera, and other sensor data and used unified timestamps to reconstruct travel paths, operating speed, header state, and grain-unloading events [128]. An operating database should therefore store parameter values together with location, time, machine identity, task type, and operating context. Yield mass-flow signals also require vibration compensation, and dual-plate differential sensing, filtering, and vibration isolation can improve the signal-to-noise ratio and reliability of yield maps [129]. A soybean-harvesting database should additionally standardize machine identifiers, field tasks, operating progress, material transfer, and fleet status to support multi-machine coordination and full-process traceability [130].

Reliable database construction also requires standardized preprocessing, spatial correction, and multi-source contextual information. Dulaney et al. constructed a yield archive covering seven crops and 43 fields from 2014 to 2021. After coordinate projection, inward boundary buffering, threshold filtering, outlier removal, and positional correction, block kriging was used to generate yield maps at a spatial resolution of 5 m [131]. Matcham et al. developed the cleanRfield package for data import, cleaning, raster conversion, and batch processing, thereby improving the standardization and reproducibility of yield-data preprocessing [132]. Remote-sensing, meteorological, terrain, and soil variables can further provide contextual information for distinguishing whether low-yield or quality anomalies originate from machine operation, crop growth, or environmental heterogeneity [133,134]. Standardized databases can also support task coordination between combine harvesters and grain-transport vehicles, although the corresponding logistics models still require validation under soybean-harvesting conditions [135].

A soybean-harvesting database can use ISOBUS/CAN systems to record header height, forward speed, cylinder speed, concave clearance, fan speed, sieve opening, and other operating parameters and associate them with timestamps, spatial coordinates, field identifiers, machines, and tasks. Integrating these records with yield maps, online quality measurements, and environmental information enables spatial diagnosis of low-yield areas and harvest-quality anomalies. Future research should standardize data formats, units, coordinate systems, timestamps, and equipment identifiers and establish procedures for quality control, anomaly labelling, calibration recording, and data-validity assessment. A complete data chain should integrate parameter acquisition, operating-state classification, yield-data cleaning, multi-source association, spatial archiving, anomaly tracing, parameter optimization, and coordinated scheduling to support harvest-quality evaluation, equipment improvement, and long-term production management.

For sensing and AI applications, the same database should also retain sensor calibration records, model versions, training or calibration domains, validation errors, uncertainty estimates, and applicable operating ranges. These metadata are necessary for traceable model updating and for preventing automatic reuse of a sensing or control model outside the conditions under which it was validated.

## 5. Multi-Crop Harvesting Equipment and Its Application to Legume Crops

### 5.1. Platform-Based Development of Multi-Crop Harvesting Equipment

**Figure 11:**
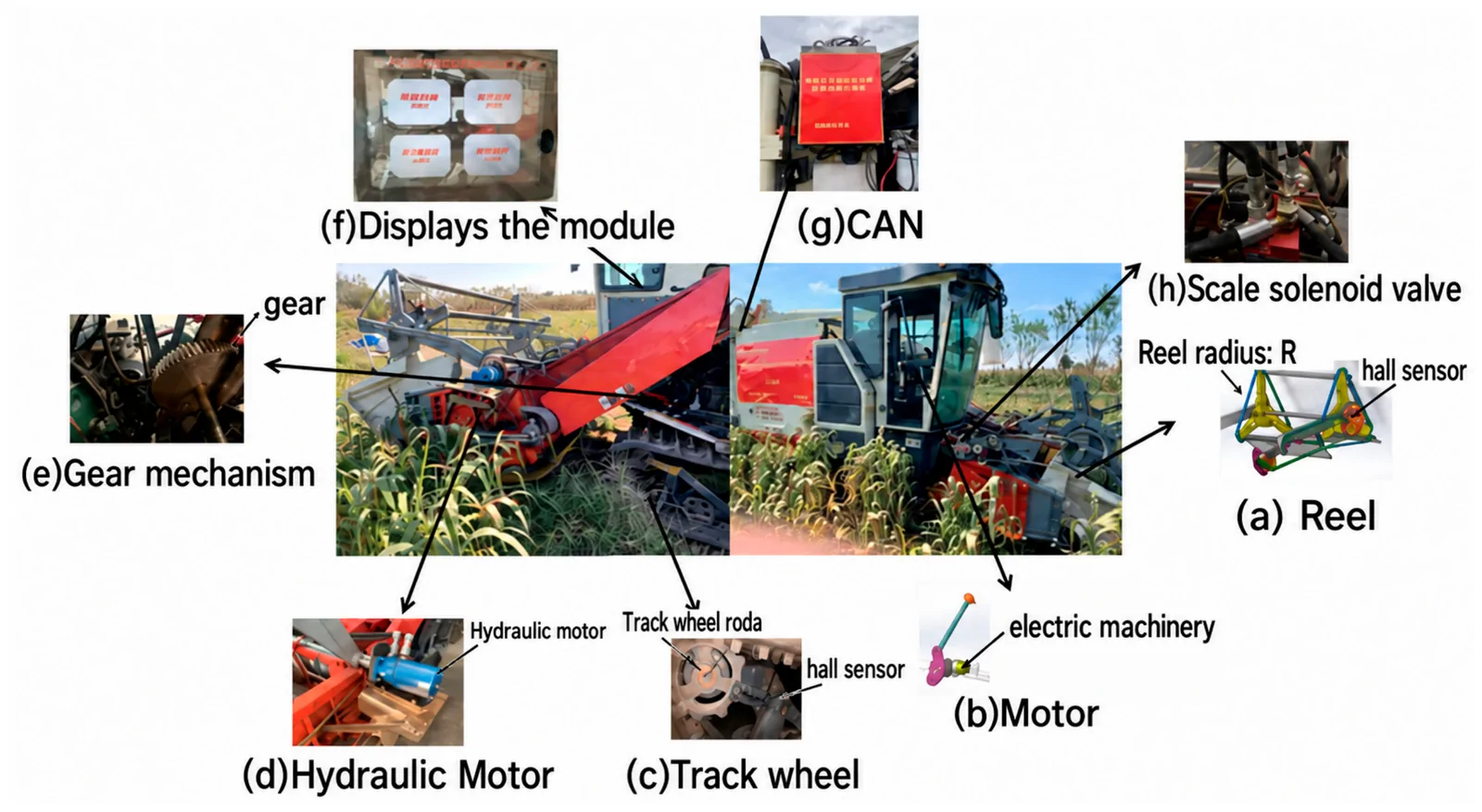
Mechanical and control components of the reel-speed and forward-speed matching system: (**a**) reel assembly; (**b**) stepper-motor actuator; (**c**) crawler drive wheel; (**d**) hydraulic motor for reel drive; (**e**) gear-transmission mechanism; (**f**) display module; (**g**) controller area network (CAN) communication module; and (**h**) electrohydraulic proportional solenoid valve. Adapted from Ji et al. [50] under the Creative Commons Attribution 4.0 International (CC BY 4.0) license. The non-English text visible in panels (**f**) and (**g**) is part of the original device labeling and does not represent scientific annotation or convey additional scientific information in this figure.

Platform-based multi-crop harvesting combines a common power chassis with crop-specific parameter sets, adjustable components, and interchangeable headers, improving annual machine utilization while reducing duplicated investment. Figure 11 shows a representative reel-speed and forward-speed matching system in which mechanical drive, sensing, communication, actuation, and control are integrated within one adjustable subsystem.

On a common multi-crop platform, automatic reel-speed matching can be implemented through sensors, electrohydraulic actuation, and fuzzy PID control [50]. Crop-specific calibration extends this concept to soybean by adjusting reel speed and installation height according to forward speed and plant condition [59]. Similar model switching can extend to threshing and cleaning through multi-parameter quality models [32]. Hardware modularity complements software adaptation: shared platforms can serve different crops by replacing selected working components and adjusting settings [136], while crop-specific modules remain necessary where row spacing, plant architecture, and feeding mode differ substantially [38].

Sensing, navigation, communication, and control modules should remain reusable across crop changes. Multi-crop boundary detection provides evidence for transferable perception algorithms [119], while modular intelligent-agriculture architectures show how CAN, ROS, and IoT communication can connect reusable functional units [137]. Generalization, transfer learning, and multimodal sensing can reduce recalibration effort across crops and machines [138]. Platform development therefore follows two complementary pathways: software-oriented crop-parameter switching and hardware-oriented rapid module replacement. Standardized communication and retrofit data interfaces can reduce integration difficulty among chassis, modules, sensors, and terminals [139]. Future multi-crop platforms should combine a common chassis, interchangeable working components, crop-parameter databases, transferable perception models, and adaptive control.

### 5.2. Representative Legume Crops and Cross-Crop Analogues

Legume crops differ in fruiting position, plant architecture, moisture condition, seed mechanical properties, and end-use requirements, so transferable harvesting technologies must be interpreted within clear applicability boundaries [1]. Soybean generally favors direct combine harvesting with close-to-ground cutting, compliant feeding, and moisture-aware threshing [1,89], and mechanical damage depends strongly on the interaction between seed moisture and threshing intensity [74]. Peanut, by contrast, requires digging, soil separation, windrowing, pickup, and pod stripping [140]. Short or lodging-prone pulse crops require stronger crop lifting and ultra-low cutting; chickpea header optimization provides a transferable example of matching reel structure to plant architecture [82]. Numerical performance should not, however, be transferred directly among crop species.

Mechanical and frictional properties also vary with crop species, moisture, loading rate, contact orientation, and harvest timing. Grain-strength studies provide transferable evidence that both moisture and loading rate influence failure behavior [141], while field measurements show that crop-surface friction can vary dynamically during harvest [142]. Fresh-market legumes require a different design emphasis because high-moisture tissues are easily bruised or abraded. A compliant comb-tooth cylinder developed for fresh vegetable soybean reduced pod damage while maintaining pod-removal performance [143]. Cross-crop comparison therefore supports differentiated machine structures, parameter ranges, and quality indicators according to fruiting position, plant architecture, material properties, and end use.

### 5.3. Modular Working Components and Operating-Parameter Databases

Modular working components and operating-parameter databases jointly determine the adaptability of combine harvesters. Adjustable threshing clearance, variable-diameter cylinders, interchangeable contact elements, and adjustable guide structures can extend the operating range of a common machine [144,145]. Variable-stiffness and compliant threshing concepts developed for maize provide transferable design references and show that component stiffness, cylinder speed, concave clearance, and moisture interact in determining damage [146,147]. For soybean, real-time feed-rate sensing can index operating-parameter databases and trigger adaptive clearance adjustment [14], while multi-input–multi-output quality models can map threshing clearance, sieve opening, and fan settings to loss, impurity, and breakage [32]. Each database entry should therefore specify crop, material state, component configuration, control variables, quality indicators, uncertainty, and validity range.

The database should be hierarchical rather than universal. Transferable modeling studies indicate that separate models may be required for different crop types, environmental states, or operating ranges [148,149], so calibration records, experimental conditions, model uncertainty, and extrapolation limits should be retained. Adjustable guide components provide another means of regulating residence time and crop flow: variable guide-plate angle can be coordinated with cylinder and forward speed to balance loss, impurity, and breakage [150], while field studies of fully adjustable guide vanes show potential benefits for throughput, damage, separation loss, and energy use [151]. Future multi-crop systems should integrate adjustable hardware, hierarchical parameter databases, state sensing, and quality feedback within one adaptive framework.

### 5.4. Harvesting Under Strip Intercropping, Hilly and Mountainous, and Small-Plot Conditions

**Figure 12:**
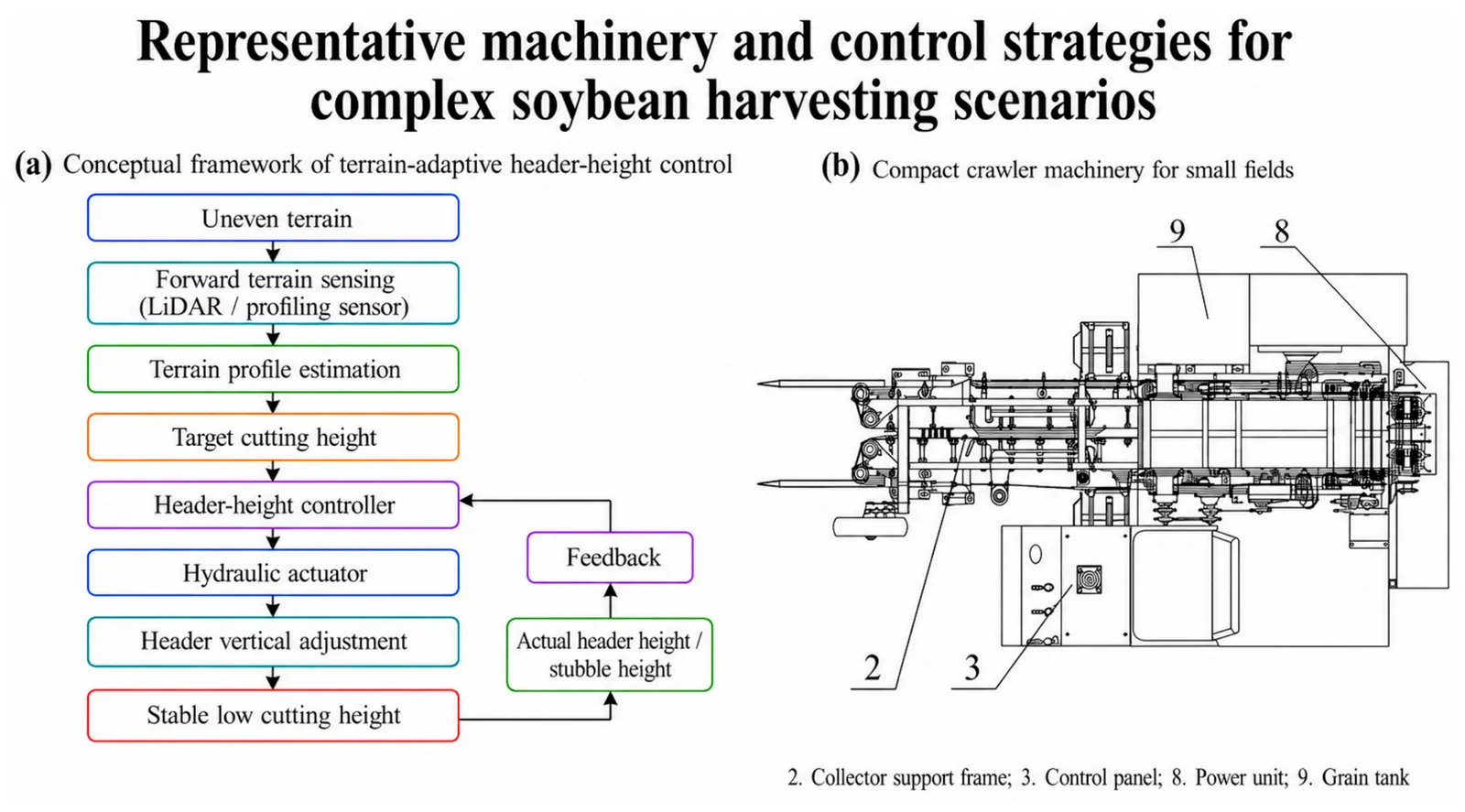
Representative machinery and control strategies for complex soybean-harvesting scenarios. (**a**) Original conceptual framework of terrain-adaptive header-height control integrating terrain sensing, state estimation, control decision, hydraulic actuation, and height feedback; and (**b**) compact crawler machinery for hilly and small-field operations. In panel (**a**), the colored outlines are used only to visually distinguish the successive functional modules in the terrain-adaptive header-height control loop and do not represent quantitative categories or levels. The technical principles in panel (**a**) are supported by Refs. [22,117], while panel (**b**) was adapted from Pan et al. [152].

Soybean equipment improves adaptability to complex production scenarios through three complementary approaches: crop-strip-aware parameter matching for intercropping, adaptive terrain-following and chassis control for uneven ground, and compact crawler-based machinery for hilly and fragmented fields. Representative strategies are summarized in Figure 12.

In maize–soybean strip intercropping, narrow rows and variable crop load increase the need for reel-speed matching and crop-specific working widths. Automatic reel regulation has reduced soybean header loss under strip conditions [59], and a crop-specific harvesting-header study addresses machinery matching to strip geometry [38]. Separately, a hooded spray system developed for soybean–maize strip intercropping [153] illustrates crop-zone protection and specialized equipment layout, but it does not demonstrate harvesting-header or loss-reduction performance. Hilly and mountainous fields require both local header terrain following and whole-machine attitude stabilization. Header systems integrating terrain sensing and hydraulic actuation can improve cutting-height consistency under uneven-ground conditions [117], while adjustable crawler chassis and robust leveling control provide complementary methods for regulating roll and pitch on slopes [154,155].

Fragmented fields additionally require compact dimensions, strong trafficability, and rapid maneuverability. Compact crawler harvesters developed for other crops provide relevant layout references [152]; plot-combine research highlights low carryover and rapid changeover [156]; and tracked-chassis studies emphasize ground clearance, track geometry, and center of gravity [157]. Reviews of hilly agricultural power chassis further support lightweight, multifunctional, and intelligent development [158]. As Figure 12 indicates, scenario adaptability therefore depends on coordinated crop-specific modules, terrain control, and chassis design rather than on a single machine configuration.

### 5.5. Interface Standards, Cost Constraints, and Conditions for Adoption

Broader deployment of intelligent harvesting equipment depends on standardized information interfaces, as well as economic viability. ISOBUS-based middleware has been developed to support data exchange and communication among agricultural implements, retrofitted machinery platforms, and external sensing and control systems [159]. High-Speed ISOBUS and OPC UA-based machinery information models extend this capability to higher-bandwidth sensing and standardized parameter exchange [160]. Multi-sensor systems also require synchronized timestamps, standardized data formats, and consistent feature definitions [161]. Economic constraints are especially important for smallholders: private combine ownership may involve long payback periods [162], while lower-cost sensing architectures can reduce the cost of intelligent functions [163]. Machinery-access models should therefore reflect farm scale and capital availability, with private ownership, outsourcing, or mixed arrangements serving different operator groups [164].

The economic feasibility of intelligent sensing also depends on the selected sensor configuration and its intended harvesting function. Camera-based systems generally offer a comparatively accessible solution for crop-row recognition and visual quality monitoring, although their practical deployment requires adequate protection against dust, vibration, and changing illumination. Three-dimensional LiDAR provides direct geometric information for crop-row reconstruction, obstacle perception, and terrain sensing, but it may involve higher equipment expenditure, computational requirements, and integration complexity. GNSS/IMU systems provide complementary positioning and attitude information, while multi-sensor fusion may improve operational robustness at the expense of additional calibration, synchronization, and maintenance requirements. Table 4 presents an indicative qualitative comparison of these configurations in terms of relative affordability, technical capabilities, integration burden, and deployment suitability [111,161,162,163,164].

A universal return-on-investment estimate cannot be derived reliably from the available studies because comparable information on system acquisition costs, installation, maintenance, annual operating hours, useful life, and monetized harvest-loss reductions is insufficient. Future techno-economic evaluations should jointly report lifecycle costs, field utilization, labor requirements, maintenance expenditure, and the economic value of verified improvements in harvest quality. For smaller farms, shared machinery services or modular retrofitting may provide more accessible implementation pathways than investment in fully integrated high-cost sensing platforms.

Training, maintenance, financing, and service organization determine whether intelligent equipment can be used sustainably. Mechanization studies identify limited awareness and training as adoption barriers [165], while service-system research emphasizes financing, maintenance capability, and value-chain coordination [166]. Maintenance training can directly reduce downtime and dependence on external support [167]. Autonomous operation adds further requirements for reliability, lifecycle cost, infrastructure, and safety, making supervised autonomy within a defined operational domain a realistic near-term pathway [168]. Technical interoperability, affordability, service capacity, and operator support should therefore be advanced together rather than treated as separate deployment issues.

## 6. Key Issues and Future Directions

### 6.1. Fundamental Harvesting Data and Material-Property Databases

A soybean-harvesting material database should integrate plant architecture, stem and pod mechanics, pod-detachment behavior, seed physical and mechanical properties, bulk-material behavior, machine-response data, and simulation parameters. Cultivar-specific differences in uprooting force, stem diameter, root length, and related plant traits show that fundamental mechanical data cannot be represented by a single generic soybean value [169]. Seed properties are also strongly influenced by cultivar, moisture, loading rate, orientation, and contact condition. Moisture alters geometry, density, friction, and angle of repose [170], while failure force, deformation, and energy absorption vary with moisture and loading rate [9]. Multifactor grain-mechanics studies in maize reinforce the need to record loading speed and orientation when interpreting failure thresholds [171], and bulk-compression studies in soybean show that layer height and viscoelastic response should also be considered [172].

The database should also connect machine settings with harvest-quality outcomes and simulation parameters. Soybean threshing studies show how element geometry can reduce damage while maintaining efficiency [90]. DEM-oriented databases require calibrated contact, bonding, and fracture parameters together with validation errors [173,174], and irregular crop geometry should be represented realistically rather than by overly simplified particles [28]. Sensor-related entries should record sensor position, calibration procedures, interference sources, and transferability across machines and crops [175]. Future work should standardize measurement conditions and preserve uncertainty, calibration records, and validity boundaries so that crop properties can be mapped traceably to equipment design, numerical simulation, sensing, and control [90,169,170].

### 6.2. Low-Loss, High-Efficiency, and Multi-Objective Coordinated Optimization

**Figure 13:**
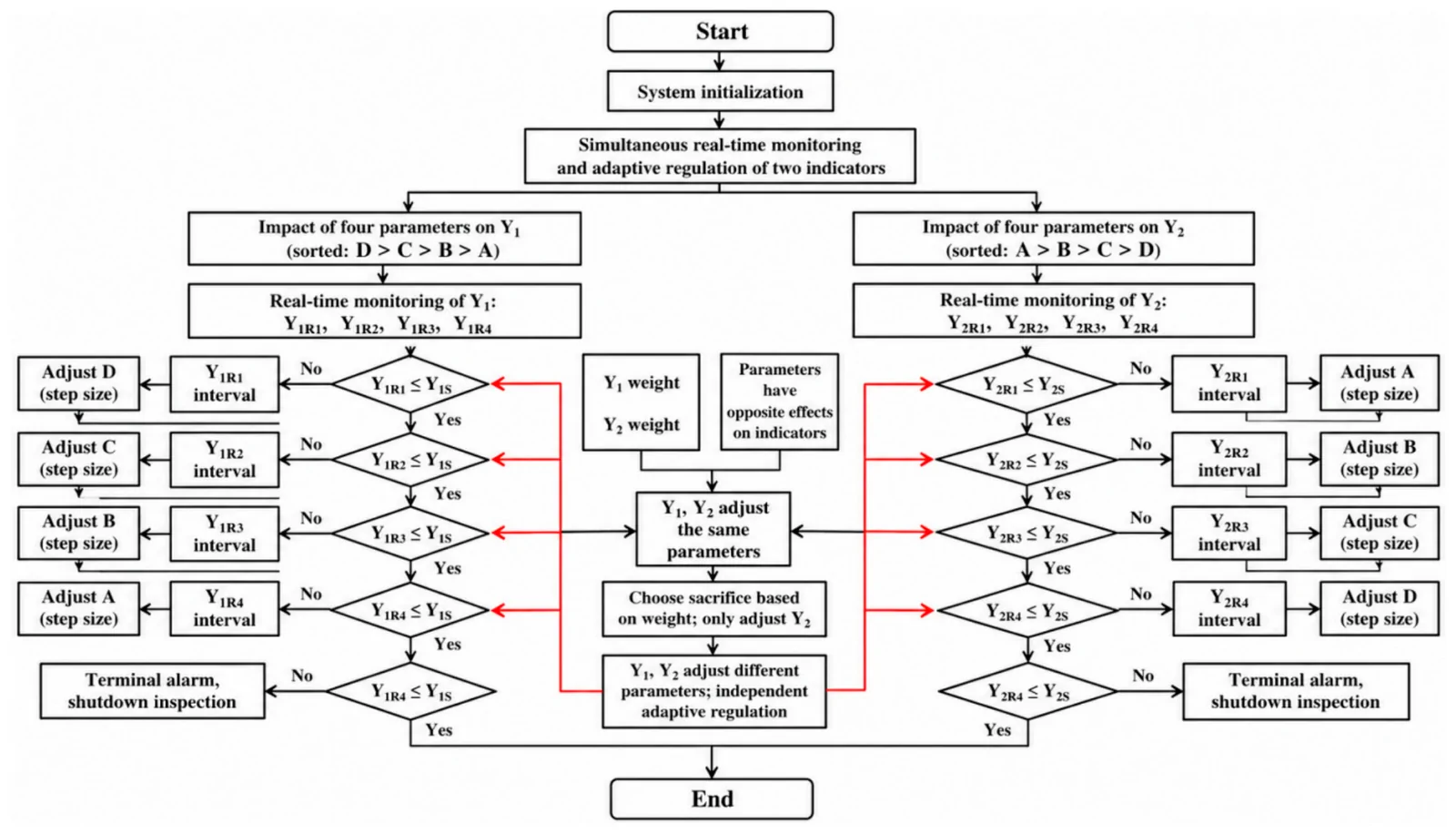
Rule-based adaptive coordination of cleaning parameters based on real-time monitoring of cleaning loss rate and impurity rate. Red arrows highlight the adaptive parameter-adjustment branches associated with the monitored cleaning-quality indicators, whereas black arrows indicate the main sequential decision flow. A, B, C, and D denote fish-scale sieve-sheet opening, damper opening, fan speed, and cleaning-screen crank speed, respectively; Y_1_ and Y_2_ denote cleaning loss rate and impurity rate, respectively.

Soybean harvesting involves unavoidable trade-offs among harvest loss, grain breakage, impurity content, unthreshed grain, throughput, energy consumption, and machine load. Threshing-element geometry and cylinder speed should therefore be optimized jointly rather than through single-parameter adjustment. Soybean tests with arc-shaped threshing teeth demonstrate that breakage can be reduced while maintaining an acceptable unthreshed-grain rate [176]. For cleaning, online loss and impurity monitoring already supports staged multi-parameter coordination in soybean [8], as summarized in Figure 13.

Rule-based control cannot fully represent nonlinear coupling and time-varying crop conditions. Feature-screening and nonlinear modeling methods developed in other sensing applications provide transferable tools for reducing redundant inputs [177], while response-surface optimization can identify parameter interactions and feasible static operating regions [178]. Online optimization can go further by updating surrogate models with recent monitoring data and solving multi-objective problems in real time; Pareto-based cleaning optimization has demonstrated this pathway [179]. Nonlinear diagnostics can guide model choice [180], and online sensing with continuous model updating provides a transferable method for compensating sensor drift and changing material condition [181].

Future multi-objective optimization for soybean harvesting should be defined as the coordinated adjustment of machine operating parameters to minimize harvest loss, grain breakage, impurity content, unthreshed grain, and energy consumption while maintaining adequate harvesting throughput and operational stability. The principal adjustable parameters include forward speed, header height, reel speed, threshing-cylinder speed, concave clearance, fan speed, and sieve opening. Their feasible adjustment ranges are constrained by crop moisture, feed rate, straw-to-grain ratio, material composition, machine power capacity, actuator operating limits, and safety requirements. These optimization objectives may conflict; for example, increasing threshing intensity may reduce the unthreshed-grain rate but increase grain breakage, while stronger cleaning airflow may reduce impurity content at the expense of greater cleaning loss. Therefore, multi-objective optimization should identify practical trade-off solutions rather than independently optimize individual performance indicators. Soybean adaptive-cleaning studies [8] and Pareto-based cleaning optimization in other crops [179] provide complementary evidence for this approach. Future research should combine real-time sensing, condition-dependent objective prioritization, and coordinated parameter adjustment, while establishing soybean-specific models and validating their performance under different field conditions.

### 6.3. Engineering Implementation of Intelligent Control Systems

Engineering implementation requires a reliable whole-machine closed loop linking sensing, decision-making, actuation, and feedback across the header, travel system, threshing and separation, and cleaning. Closed-loop architectures developed in other agricultural processes highlight model reliability, real-time sensing cost, and equipment stability as engineering constraints [182]. For combine harvesters, multi-parameter threshing-and-cleaning models combined with predictive control demonstrate the feasibility of coordinated regulation [183]. Separately, sensor-based fuzzy modeling of disc–soil forces has been demonstrated in soil-bin tillage experiments [184], providing only a methodological analogy for possible header–ground contact-load estimation. It does not validate soybean feed-rate prediction or harvest-quality control. Whole-machine intelligence also requires terrain-following headers, adaptive threshing, navigation, and preharvest decision-making to operate coherently. Multi-sensor predictive header control can compensate for terrain and actuator delay [185], feed-rate sensing can drive threshing-clearance adjustment [14], adaptive path tracking can improve travel accuracy and effective header utilization [186], and UAV-derived prescription maps provide a transferable route for presetting expected crop load by field zone [187].

Real-time feasibility must be evaluated at the complete-system level rather than inferred from a single algorithm benchmark. Fang et al. reported approximately 122.7 ms of processing per frame for mature-soybean row detection [111], which is not the total time from acquisition to steering or hydraulic movement. End-to-end delay also includes sensing, filtering or fusion, communication, controller scheduling, and actuator response; harvest-quality feedback is further affected by crop-material transport time. Embedded evaluations should identify hardware configuration and report sampling frequency, typical and worst-case latency, timing jitter, missed deadlines, communication losses, and the response to sensor dropout or peak computational load. An acceptable control deadline depends on machine speed, look-ahead distance, actuator bandwidth, and material residence time rather than on a universal numerical threshold. Hardware-in-the-loop tests and synchronized field measurements would help determine the feasible operating envelope for delay-aware adaptive regulation [113,116,185].

Communication reliability is essential for coordinated sensing, decision-making, and actuation in intelligent soybean combine harvesters. Onboard CAN/ISOBUS networks and remote communication links may experience transmission delays, message loss, outdated data, or temporary disconnections, potentially affecting the accuracy and timeliness of control decisions. Reliable system implementation should therefore incorporate message timestamps, data-freshness checks, heartbeat monitoring, communication timeout detection, and fault logging. Time-critical control functions, including header-height regulation, travel control, and threshing and cleaning adjustments, should remain executable by onboard controllers without continuous dependence on wireless or cloud connectivity. Future evaluations should consider communication latency, timing jitter, packet loss, recovery time, and the effects of communication interruptions on closed-loop control performance.

Cybersecurity represents another important requirement for interconnected harvesting machinery. Brodie and Oksanen investigated cybersecurity issues associated with CAN-based ISO 11783 communication and evaluated the use of OPC UA for secure communication in agricultural vehicles [188].de a comprehensive cybersecurity framework. Potential threats include unauthorized access, forged control commands, manipulation of transmitted data, and compromised software updates. Appropriate protection measures include device authentication, message-integrity verification, access control, secure software updating, and network anomaly detection. The communication overhead associated with such security mechanisms should also be considered in system implementation [188]. However, the effectiveness, computational requirements, and real-time compatibility of these security measures still require validation in soybean-harvesting environments.

Fault detection and degraded-mode operation should also be incorporated into the whole-machine control architecture. When sensor measurements become unreliable or communication is temporarily interrupted, the controller should identify abnormal conditions, provide operator warnings, and adopt predefined fallback strategies according to the severity of the fault. Where supported by a machine-specific safety assessment, temporary operation with conservative parameter settings may be permitted; persistent or safety-critical faults should trigger suspension of affected automatic functions, operator intervention, or a controlled stop when necessary. Fault prediction and fuzzy speed control provide approaches for identifying blockage risks and reducing machine speed before severe overload develops [189], while edge-cloud monitoring supports remote diagnostics and maintenance [190]. Digital twins offer additional possibilities for virtual commissioning, state visualization, performance prediction, and preventive maintenance. Existing combine-harvester studies demonstrate physical–virtual synchronization [191], while agricultural machinery reviews and transferable digital-twin control studies provide references for condition monitoring and predictive regulation [192,193]. Future research should integrate communication reliability, cybersecurity, fault-tolerant control, and long-duration field validation into the engineering evaluation of intelligent soybean-harvesting systems.

### 6.4. Modular Interfaces and Regionalized Deployment Pathways

Reconfigurable harvesting platforms require both modular mechanical hardware and standardized information interfaces [194,195]. Common chassis with interchangeable task modules can reduce duplicated investment and conversion time [196], while multi-task agricultural workcells show that module replacement must be coordinated by a unified controller capable of loading the correct parameters and action sequence [197]. True plug-and-play operation therefore requires joint standardization of mechanical, power, hydraulic, electrical, and information interfaces. Standardized communication provides an important information foundation for modular agricultural machinery. Paraforos et al. reviewed ISO 11783-compatible sensor and control systems for agricultural machinery, including their applications in data acquisition, transfer, monitoring, and control [195]. High-Speed ISOBUS and OPC UA-based information models provide greater bandwidth and standardized device descriptions [160], shared sensing and multitask models can reduce hardware duplication [198], and middleware can integrate legacy equipment without native ISOBUS capability [159].

Regional deployment must match equipment packages and service models to local crop combinations, harvest calendars, field scale, terrain, financing, maintenance capability, and operator training. Mechanization-service studies show that centralized service provision can reduce acquisition barriers but remains constrained by financing and maintenance capacity [166]. Regional agricultural programs likewise require coordination among manufacturers, service providers, financial institutions, and other stakeholders [199]. Future deployment should therefore combine modular equipment, standardized interfaces, validated parameter databases, and professional service networks rather than rely on whole-machine sales alone [159,194,195]. Recent reviews of grain-combine control algorithms further indicate that whole-machine intelligence requires coordinated sensing, decision-making, and multi-parameter regulation rather than isolated controller design [200].

### 6.5. Conclusions

Mechanized soybean-harvesting quality is jointly determined by crop characteristics, planting systems, equipment structures, operating parameters, field environments, and management. Low pod position, lodging, pod shattering, seed fragility, and moisture variation remain major causes of header loss, mechanical damage, and unstable threshing and cleaning. The reviewed literature shows a transition from isolated component optimization toward online sensing, machine vision, LiDAR, multi-source fusion, and coordinated adaptive control. However, threshing and cleaning remain nonlinear, time-varying, delayed, and strongly coupled, and most intelligent systems have been validated under limited cultivars, machine configurations, or field conditions.

Future development should integrate crop and terrain sensing, feed-rate estimation, header regulation, navigation, threshing, cleaning, and grain-quality monitoring within a whole-machine sensing–decision–actuation–feedback architecture. Priority should be given to robust multi-source sensing, field-calibrated state estimation, multi-objective coordinated control, standardized operating and quality databases, interoperable interfaces, fail-safe operation, and cross-scenario validation. Regional deployment should also consider farm scale, financing, maintenance, service capacity, and operator training. Representative soybean studies reviewed here reported reductions of approximately 30.4% in grain breakage, 62.6% in harvest loss, and 50.0% in cleaning loss under their respective experimental conditions, demonstrating the potential of sensing-guided adaptive regulation while underscoring the need for broader field validation.

## Figures and Tables

**Table 1:** Use-oriented quality-control indicators, harvesting risks, and machinery-control priorities for soybean.

End-Use Category	Main Quality-Control Indicators	Primary Harvesting Risks	Key Control Parameters/Strategies	Main Equipment Stages	Evidence Basis
Commercial grain soybean	Total harvest loss; header loss; threshing and cleaning loss; grain breakage; impurity content; commercial grade; storage safety	Missed pods and field loss; header impact loss; split and cracked seeds; excessive pod and stem impurities; poor cleaning performance	Header height; forward speed; reel speed; drum speed; concave clearance; fan speed; sieve opening; coordinated loss–breakage–impurity optimization	Header; threshing/separation; cleaning	[8,11,49]
Seed soybean	Mechanical damage; seed-coat cracking; germination; seed vigor; field emergence; sanitary quality	Invisible internal damage; delayed-harvest deterioration; fungal contamination; reduced germination and vigor	Timely harvesting; gentle threshing; low-impact conveying; reduced drop height; careful grain handling; avoidance of excessive impact and compression	Harvest timing; threshing; conveying/handling	[6,42,43]
Food-processing soybean	Grain integrity; size uniformity; seed-coat integrity; color and cleanliness; low impurity; processing suitability	Breakage affecting appearance and processing quality; contamination; non-uniform size; reduced batch consistency	Gentle threshing; fine cleaning; low-impact conveying; size grading; batch segregation and uniform handling	Threshing; cleaning; conveying; grading	[23,46]
Fresh vegetable soybean	Pod integrity; pod appearance; low cracking and shattering; low abrasion and bruising; market quality	Pod cracking; surface abrasion; mechanical bruising; appearance deterioration; mixed damaged pods	Timely harvesting; flexible low-impact pickup; gentle conveying; careful pod handling; sorting and grading	Harvest timing; header/feeding; conveying/handling; grading	[47]

Note: This table is a qualitative synthesis for Section 2.3. Cross-study numerical ranking is not performed, because crop type, machine configuration, moisture condition, and evaluation methods differ among studies. Nutritional and flavor-related traits are end-use attributes rather than direct harvester-control outputs.

**Table 2:** Process routes and applicable conditions of manual cutting, two-stage harvesting, and direct combine harvesting of soybean.

Comparison Dimension	Manual Cutting and Stationary Threshing	Mechanized Windrowing and Pickup	Direct Combine Harvesting
Process route	Close-to-ground manual cutting → bundling or laying → natural drying → stationary threshing	Mechanized windrowing → field drying → pickup feeding → threshing and cleaning	Header cutting → feeding → threshing and separation → cleaning in one continuous pass
Material-state change	Standing crop is manually cut and converted into bundles or loose whole plants, which are dried before threshing.	Standing crop is converted into an aligned windrow; field drying reduces moisture before pickup and threshing.	Standing plants enter the combine directly and are continuously converted into cleaned grain and residues.
Operating efficiency	Low; operation is discontinuous and involves multiple handling steps.	Moderate; field drying separates cutting from pickup and threshing.	High; cutting, feeding, threshing, separation, and cleaning are completed in one pass.
Labor input	High; substantial manual cutting, handling, transport, and feeding are required.	Moderate; mechanized cutting and pickup reduce labor demand, but an additional field operation is required.	Low; labor is mainly required for machine operation, adjustment, grain transport, and logistics.
Weather dependence	High; drying duration and repeated handling are strongly affected by rainfall and ambient humidity.	Moderate to high; successful field drying requires a suitable weather window and timely pickup.	Moderate; exposure after cutting is limited, but harvest must occur within a suitable maturity and moisture window.
Quality protection	Carefully managed cutting, drying, and stationary threshing can protect seed vigor and reduce impact damage.	Suitable for high-moisture or unevenly mature crops; field drying can improve threshability, but rewetting and windrow shattering must be controlled.	Good quality can be achieved when crop characteristics, grain moisture, header operation, threshing intensity, and cleaning settings are appropriately matched.
Principal harvesting risks	Missed low pods; contamination during field drying; deterioration or mold under delayed drying; loss during repeated handling.	Windrow shattering; pickup loss; rewetting; uneven windrow density; soil or foreign-material contamination.	Header loss; pod shattering; feeding blockage; grain breakage; incomplete threshing; excessive impurity content.
Key control priorities	Low and stable cutting height; careful bundling and transport; clean drying surfaces; gentle stationary threshing.	Uniform windrow formation; moisture monitoring; timely pickup; low-impact pickup feeding; coordinated forward and pickup speeds.	Header height; forward speed and reel speed; feeding continuity; cylinder or rotor speed; concave clearance; fan and sieve settings.
Applicable crop and field conditions	Small or fragmented fields; lodged or difficult crops; limited machine access; quality-sensitive seed lots where labor is available.	High-moisture crops; uneven maturity; quality-sensitive harvests; fields suitable for windrowing with reliable drying conditions.	Uniform maturity; suitable grain moisture; relatively upright crops; accessible fields; crop structure compatible with continuous feeding.
Main equipment and stages	Hand tools or small cutters; drying area; stationary thresher; separate cleaning or grading equipment.	Windrower or cutting platform; pickup header; threshing, separation, and cleaning unit.	Combine header; feeding system; threshing and separation system; cleaning system; grain collection and unloading.
Overall technical position	High flexibility/low efficiency.	Intermediate flexibility and efficiency.	High efficiency/strict crop-suitability requirements.

Note: The comparison is qualitative and is intended to define the technical boundaries of each harvesting route rather than rank the routes using unverified numerical scores.

**Table 3:** Quantitative comparison of representative sensing, monitoring, and adaptive-control studies relevant to soybean harvesting.

Study/Crop	Method and Target	Validation/Trials	Key Results	Evidence Type	Principal Limitation
Jin et al. [13] Soybean	Improved U-Net grain imaging; broken grains/impurities	Bench: 120 images (110 train, 10 test); three 30-kg batches	F1: Intact 93.04%, broken 89.40%, impurity 96.49%; max rate errors 0.57/0.69 percentage points	Direct soybean (D1)	Single cultivar; no long-term on-machine validation
Liu et al. [8] Soybean	Piezoelectric sensing + fuzzy air–sieve control; loss/impurity	Field: 2 cultivars (14.9–15.2% moisture); 16 samples each; 17 verification sets	Cleaning loss 0.38% → 0.19%; impurity 2.66% → 1.68%	Direct soybean (D1)	Short trials; wider operating range unverified
Liu et al. [14] Soybean	Vision, header height and moisture; PSO–BP feed-rate estimation/adaptive concave	Soybean field vs. fixed clearance; independent repetitions NR	Feed-rate accuracy 90.8%; breakage 3.39% → 2.36%; loss 1.87% → 0.70%	Direct soybean (D1)	Specific field/machine; latency and multi-season transfer NR
Nie et al. [22] Soybean	Mechanical terrain following + compliant reel/conveyor; header loss/stubble	Field: Nandou 25 (14.7% moisture); 3 × 20-m runs; 50 stubble points	Header loss 1.34% (55% below rigid header); stubble 70.36 mm (22.7% lower)	Direct soybean (D1)	Limited cultivar coverage; no electronic closed loop
Leng et al. [59] Soybean	PLC reel-speed control; speed tracking/header loss	Soybean–maize strips: 6 speed tests; 15 controlled/13 traditional groups; 3 optimal-setting repeats	Max speed error 5.38%; best losses 3.85% (traditional) vs. 1.73% (controlled): 2.12 percentage points difference	Direct soybean (D1)	Compared different optimal settings; limited strips
Zhang et al. [100] Soybean	Piezoelectric plate + FFT/threshold counting; cleaning loss	Mixed-material lab tests (3 repeats); soybean field (5 tests)	Grain recognition up to 95.1%; field loss-rate relative error < 4%	Direct soybean (D1)	Field calibration; limited machine transfer
Fang et al. [111] Mature soybean	3D LiDAR stem extraction/point-cloud partition; row centerline/heading	Mature-soybean field: 3 planting densities; scan count NR	Centerline accuracy 90.6%; angular error 2.35°; 122.7 ms/frame	Soybean support (D2)	Perception only; closed-loop field validation absent
Ou et al. [118] Soybean–maize	3D LiDAR clustering/line fitting; crop-row navigation	Strip field: missing rows, low/high occlusion; 100 frames per scenario	Line accuracy > 80% (up to 90% at low occlusion); mean angle within 0.28°; <75.62 ms/frame	Soybean support (D2)	Navigation only; end-to-end actuator latency NR
Golpira and Sola-Guirado [82] Chickpea	Fuzzy loss model + simulated annealing; reel design/header loss	5 prototype reels; 3 field sites (2007–2017) and virtual optimization	Optimized reel harvested ~71% of pods; losses 47–29% across prototype years	Adjacent legume	Offline design; different prototype years; no soybean test
Chen et al. [101] Rice	Camera/color classification; broken-grain recognition	Rice laboratory evaluation: ~2300 grains	Grain-breakage recognition accuracy 96%	Cross-crop transfer	Rice laboratory only; no soybean/field testing
Lee et al. [120] Rice	Dual-GPS navigation + alpha-shaped turns; path tracking	Rice-combine field trials, with/without harvesting; repeats NR	During harvest: mean errors 0.038 m (straight), 0.195 m (turn)	Cross-crop transfer	Transfer to mature-soybean conditions untested
Yu et al. [124] Maize	Piezoelectric loss sensor + fuzzy-neural PID; concave clearance	Maize field: 3 speeds; control on/off at medium speed	Entrainment loss 43.75% lower with control at medium speed	Cross-crop transfer	No soybean quality or delay validation
Chai et al. [125] Rapeseed	Piezoelectric loss sensor + grey prediction/fuzzy sieve control	Rapeseed field: 3 speeds; controller on/off, 3 repeats each	Loss within control band; RMSE up to 49.92% lower; paired mean-loss reduction NR	Cross-crop transfer	Rapeseed only; soybean transfer/full delay not validated

Note: NR = not reported. Performance values apply to the original crop, machine, and test conditions; transfer studies are not direct soybean-harvesting validation. Frame-processing time is not complete sensor-to-actuator response time. Where available, field duration, number of trials, cultivar information, moisture conditions, machine configuration, and statistical significance were extracted from the original studies. Information not reported in the source publications was treated as a reporting limitation and was not inferred.

**Table 4:** Indicative techno-economic comparison of sensing configurations for soybean-harvesting applications.

Sensing Configuration	Relative Affordability	Principal Benefits and Limitations	Suitable Deployment Context
RGB camera	Generally more accessible; additional image-processing and protection costs	Crop-row and grain-image recognition; sensitive to illumination, dust, and occlusion	Cost-sensitive sensing retrofits and basic monitoring
3D LiDAR	Generally higher equipment and processing burden	Three-dimensional crop and terrain information; calibration, dust, and data-processing challenges	Complex-field perception where geometric information is important
GNSS/IMU	Configuration-dependent; correction services may add recurring costs	Position and attitude estimation; does not independently resolve crop condition or grain quality	Navigation and machine-state monitoring
Multi-sensor fusion	Usually greater integration and maintenance burden	Complementary information and potential redundancy; synchronization and calibration complexity	Highly utilized machines requiring multiple sensing functions

Note: The comparison is qualitative and reflects indicative engineering considerations rather than measured market-price differences or experimentally verified investment returns. Actual costs and adoption suitability depend on sensor specifications, farm scale, annual machine utilization, local service conditions, and system configuration.

## Data Availability

The literature data used in this review were obtained from the databases and sources described in Section 1.4. No new experimental datasets were generated in this study.
